# Endocytic recycling protein EHD1 regulates primary cilia morphogenesis and SHH signaling during neural tube development

**DOI:** 10.1038/srep20727

**Published:** 2016-02-17

**Authors:** Sohinee Bhattacharyya, Mark A Rainey, Priyanka Arya, Samikshan Dutta, Manju George, Matthew D. Storck, Rodney D. McComb, David Muirhead, Gordon L. Todd, Karen Gould, Kaustubh Datta, Janee Gelineau-van Waes, Vimla Band, Hamid Band

**Affiliations:** 1 The Department of Pathology & Microbiology, University of Nebraska Medical Center, Omaha, NE, USA; 2 The Department of Genetics, Cell Biology & Anatomy, University of Nebraska Medical Center, Omaha, NE, USA; 3 The Department of Biochemistry & Molecular Biology, University of Nebraska Medical Center, Omaha, NE, USA; 4 Eppley Institute for Research in Cancer and Allied Diseases,University of Nebraska Medical Center, Omaha, NE, USA; 5 Fred and Pamela Buffett Cancer Center, University of Nebraska Medical Center, Omaha, NE, USA; 6 Department of Pharmacology, Creighton University School of Medicine, Omaha, NE, USA

## Abstract

Members of the four-member C-terminal EPS15-Homology Domain-containing (EHD) protein family play crucial roles in endocytic recycling of cell surface receptors from endosomes to the plasma membrane. In this study, we show that *Ehd1* gene knockout in mice on a predominantly B6 background is embryonic lethal. *Ehd1*-null embryos die at mid-gestation with a failure to complete key developmental processes including neural tube closure, axial turning and patterning of the neural tube. We found that *Ehd1*-null embryos display short and stubby cilia on the developing neuroepithelium at embryonic day 9.5 (E9.5). Loss of EHD1 also deregulates the ciliary SHH signaling with *Ehd1*-null embryos displaying features indicative of increased SHH signaling, including a significant downregulation in the formation of the GLI3 repressor and increase in the ventral neuronal markers specified by SHH. Using *Ehd1*-null MEFS we found that EHD1 protein co-localizes with the SHH receptor Smoothened in the primary cilia upon ligand stimulation. Under the same conditions, EHD1 was shown to co-traffic with Smoothened into the developing primary cilia and we identify EHD1 as a direct binding partner of Smoothened. Overall, our studies identify the endocytic recycling regulator EHD1 as a novel regulator of the primary cilium-associated trafficking of Smoothened and Hedgehog signaling.

Endocytic traffic of plasma membrane proteins and lipids is a basic process that controls diverse cellular processes such as nutrient uptake, cell polarity, signaling, adhesion, ion transport and neurotransmission^1^,^2^,^3^,^4^,^5^,^6^. Basal or stimulus-elicited internalization of plasma membrane components occurs by clathrin-dependent or clathrin-independent routes^2^. Internalized membrane receptors may traffic into lysosomes where they are degraded, as seen with many growth factor-stimulated signaling receptors^7^. Most internalized receptors, however, are recycled back to the cell surface, either to the original plasma membrane domain from where they were endocytosed, or to a different domain of the plasma membrane^4^,^8^,^9^,^10^. Endocytic recycling plays a critical role in returning the bulk membrane components to the plasma membrane, as well as during processes where new plasma membrane is targeted to specific destinations, such as cytokinesis^8^.

The four members of the mammalian EHD protein family (EHD1-4) are highly conserved homologs of the *C. elegans* RME-1 protein identified in a screen for mutants that impair endocytic traffic across the gut epithelium into coelom^11^. EHD proteins are characterized by an N-terminal helical domain, an ATP-binding G domain, another helical domain, a linker region and a C-terminal Eps15-homology (EH) domain. Crystal structure of EHD2 has revealed it to be a dimer whose G-domain folds similar to the GTPase domain of dynamin, suggesting that EHD proteins play roles in vesiculation analogous to dynamin^12^. Recent work has also suggested that EHD proteins may cooperate with dynamin in vesiculation^12^,^13^,^14^,^15^. The EH domain-mediated interactions with proteins containing Asn-Pro-Phe (NPF) or related peptide motifs and the ability of EHD proteins to bind to phosphatidylinositol 4,5-bisphosphate [PI(4,5)P2] are also critical for their function in receptor traffic, as shown by cell-based studies^16^,^17^. Indeed, a number of identified EHD-binding proteins such as Rabenosyn-5, SNAP29/GS32, Syndapin I and II, α-adaptin subunit of AP2, Rab11-FIP2, EHBP1 and the Ferlin proteins are involved in endocytic traffic^18^,^19^,^20^,^21^,^22^.

Mammalian EHD1 is by far the most studied of the EHD protein family in cell-based studies, which have shown its role in facilitating endocytic recycling of MHC-I, AMPA receptors, insulin-like growth factor 1 receptor (IGF1R), insulin-responsive glucose transporter 4 (GLUT4) and transferrin receptor^22^,^23^,^24^,^25^,^26^,^27^,^28^. While these studies clearly support the roles of EHD1 protein as a pivotal player in endocytic recycling, much less is known about the functions of EHD1 in regulating *in vivo* physiological processes. To investigate the *in vivo* functional roles of mammalian EHD proteins, we and others have recently employed a gene knockout approach. Deletion of mouse *Ehd1* on a 129Sv/Ev genetic background was reported in one study to have no phenotypic impact^29^. Using a different targeting strategy (exon 1 in our studies versus part of exon 3 and 5 and all of exon 4 in the Rappaport study) and a different genetic background (mixed 129/B6), however, we showed that *Ehd1* deletion leads to partial embryonic loss and male infertility due to abnormal spermatogenesis among those mice that were born^30^. *Ehd4* deletion did not have any impact on development but led to reduced testis size with moderate reduction in sperm count and male fertility^31^. While deletion of EHD3 had no obvious impact, concurrent deletion of *Ehd3* and *Ehd4* led to early death of live-born pups, which exhibited renal thrombotic microangiopathy^32^. The gross phenotypes of knockout models suggest considerable redundancy, with the exception of *Ehd1* whose deletion appeared to have a substantial genetic background-dependent impact. For example, EHD4 was identified as a Cadherin 23 interactor in the inner ear hair cells and these proteins co-localized at the tissue level; however, EHD4-null mice were functionally normal, apparently reflecting compensation by EHD1 whose expression increased in EHD4-null inner ear hair cells^33^. Further analyses of the knockout mouse models we have generated have begun to reveal roles of EHD proteins in other key physiological functions. For example, EHD3-deficient mice display structural and functional defects in heart, including bradycardia and rate variability, conduction block, and blunted response to adrenergic stimulation^34^,^35^,^36^. These defects were associated with impaired trafficking of Na/Ca exchanger and L-type Ca channel type 1.2 to the plasma membrane in EHD3-deficient myocytes, with a parallel reduction in Na/Ca exchanger–mediated membrane current and Cav1.2-mediated membrane current^34^,^35^,^36^. These results are consistent with EHD3 interaction and co-localization with Ankyrin B, a protein required for membrane targeting and stability of ion channels in cardiomyocytes, and upregulation of EHD3 levels during cardiac ischemia and failure^34^,^35^,^36^. Recently, *Ehd1*-null mice were demonstrated to have smaller skeletal muscle fibers^37^, consistent with the interaction of EHD proteins with ferlins in regulating myocyte proliferation and fusion *in vitro*^38^.

Given the key physiological roles of EHD1 revealed by *in vivo* studies thus far, and evidence from culture models that EHD1 has the potential to regulate recycling of a number of surface receptors, we have investigated its role in murine embryonic development using a predominantly B6 background in which we find EHD1 deletion to be embryonic lethal. These studies reveal a novel and critical role of EHD1 in orchestrating neural tube development by regulating primary cilia morphogenesis and SHH signaling.

## Materials and Methods

### Generation of *Ehd1* gene-targeted mice

All experiments involving animals were approved by the University of Nebraska Medical Center Institutional Animal Care and Use Committee and carried out under the approved IACUC protocol. All animals were treated humanely in accordance with institutional guidelines and that of the National Institutes of Health (NIH) Guide for the Care and Use of Laboratory Animals. Generation of *Ehd1* gene-targeted mice has been described previously^30^. To generate the mice used in this study, Cre transgene-negative *Ehd1*^+/−^ mice were backcrossed with C57BL/6J mice (The Jackson Laboratory) for five generations and the genetic background was analyzed using Illumina Golden Gate genotyping assay to examine single nucleotide polymorphisms throughout the mouse genome (DartMouse Speed Congenic facility at Dartmouth Medical School).

### Embryo preparation

For all embryonic studies, timed pregnancies were set up between *Ehd1*^+/−^ males and *Ehd1*^+/−^ females and vaginal plugs checked the next morning to establish the day of conception. Noon of the day of a positive vaginal plus was considered E0.5. At the specified times, pregnant females were sacrificed by regulated CO_2_ inhalation, the abdominal cavity was opened and the uterine horns were removed and placed into a dish containing chilled PBS. The decidua was dissected to remove the individual embryos, which were fixed in 4% paraformaldehyde, while the surrounding yolk sacs were individually saved for genotyping. After fixation for 24 hours at 4 °C, the embryos are washed in PBS, dehydrated through a graded series of ethanol washes, cleared in xylenes and embedded in paraffin. Sections were cut at a thickness of 4 μ and mounted onto slides treated for attachment.

### Genotyping

Mouse tail and embryonic yolk-sac DNA was extracted according to the vendor protocol (Gentra Puregene Mouse Tail Kit, Qiagen catalog #158267) and hydrated in water. PCR products corresponding to various *Ehd1* alleles were amplified in a duplex PCR reaction with 3 primers (primers 1–3), as described previously^30^, and separated on 2% agarose gels. Primer Sequence 1: 5′-AAGTCAGAAGACAACTTTCTGGAGTTCCCT-3′, Primer sequence 2: 5′-TCCAGGGCCCACATGGTAGAAGGAGAGAGT-3′, primer sequence 3: 5′-GCTCCGGTCTTGGACTTCACCAGCATTTAG-3′. The product are: *Ehd1* WT allele-403 bp product with primers 1 and 2; *Ehd1*-null allele-305 bp product with primers 2 and 3.

### Antibodies, Plasmids and Reagents

Antibodies were obtained from the following sources: Rabbit polyclonal anti-EHD1, anti-EHD2, anti-EHD3 and anti-EHD4 antibodies, generated in our laboratory have been described previously^27^. The antibody generated against a synthetic EHD1 peptide (amino acids 519–534: CADLPPHLVPPSKRRHE) was cross-reactive with EHD1 and EHD4 and was used to immuno-blot EHD1 and EHD4 as described previously^27^,^30^. Anti-dynamin 1 (#610245), anti clathrin heavy chain(# 610499) and anti AP-2 (#610381) antibodies were from BD; anti-Pacsin2 (#AP8088b) and anti-Epsin2 (#AP2182a-ev)were from Abgent; anti-Epsin1(#sc-48372) and anti-SHH (#sc9024) was from Santa Cruz Biotechnology; anti-myoferlin(#HPA014245),anti- acetylated tubulin(#T7451) and anti-gamma tubulin(#T6557) antibodies were from Sigma, anti-dysferlin (#ab124684) and Pericentrin antibodies were from Abcam (#ab84542),anti-GLI1 antibody was from Cell Signaling(#L4B210) and anti-GLI3 antibody was from R&D systems(#AF3690). Neural patterning analysis was performed with the following antibodies: mouse anti-NKX6.1 [Developmental Studies Hybridoma Bank (DSHB)], mouse anti-PAX6 (DSHB), mouse anti-PAX7 (DSHB), mouse anti-FOXA2 (DSHB), mouse anti-NKX2.2 (DSHB). Anti-Arl13B was from the NIH NeuroMab facility at UC Davis. For proliferation and apoptosis assays we used the rabbit IgG anti-phospho-histone H3 (Millipore) and anti-cleaved caspase 3 (Cell Signaling), respectively. The polyclonal goat anti-PECAM-1 antibody was from Santa Cruz Biotechnology. The anti GLI2 antibody was a generous gift from Dr. Jonathan Eggenschwiler, Columbia University^39^. Anti-Smoothened antibody was a generous gift from Dr. Kathryn Anderson, Memorial Sloan-Kettering Cancer Center and Dr. Raj Rohatgi, Stanford University. Alexa 488-transferrin and Alexa 488 and 633 secondary antibodies were from Invitrogen. Adenovirus was from the Viral Vector Core at the University of Iowa.

### Cell culture

As Embryonic Fibroblasts (MEFS) derived from EHD1-null embryos could not be maintained in culture for more than three passages, we established MEFs from individual E9.5 *Ehd1* floxed/floxed embryos using standard protocols (Ocbina and Anderson, 2008; Hoover *et al.*, 2008; Svard *et al.*, 2006) and cultured these in High Glucose DMEM, 0.05 mg/ml Penicillin, 0.05 mg/ml Streptomycin, 2 mM L-Glutamine, and 10% Fetal Bovine Serum (FBS) at 37 °C and 5% CO2. The *Ehd1* floxed/floxed MEFs were then rendered *Ehd1*^−/−^ by infection with Adenovirus particles expressing Cre recombinase and a GFP reporter. GFP positive cells were FACS sorted for further analysis. The genotypes of floxed/floxed vs. *Ehd1*^−/−^ MEFS were determined by analysis of lysates of confluent cell monolayers by polymerase chain reaction (PCR) and immunoblotting.

For analysis of primary cilia, confluent MEF cultures were shifted from 10% to 0.5% FBS 24–48h after plating to induce ciliogenesis and treated with 100nM Smoothened agonist (SAG; Calbiochem 566660) to activate the SHH pathway. NIH3T3 SMO-GFP cells were generated by transfecting NIH3T3 cells with a SMO-GFP reporter construct (Genecopoeia #MPRM17869-LvPF02) and selecting stable clones after FACS sorting.

### Immunofluorescence of mouse tissue sections

Fixed tissue sections were incubated overnight at 4 °C with the primary antibody, and the DAPI staining was performed for 10 min in PBS after the secondary antibody incubation followed by mounting with Prolong Gold (Life Technologies, USA).

### Quantitative PCR (QRT-PCR) Analysis of Gene Expression in Embryos

Total RNA was extracted from individual E9.0–E9.5 embryos using standard techniques, quantified using NanoDrop ND-1000 (Thermo Scientific) and cDNA was prepared from 2 μg RNA using oligo(dT) and SuperScript III reverse transcriptase (Invitrogen), as described by the manufacturer. QRT-PCRs were performed using SYBR Green-based reactions on a CFX96 system from Bio-Rad. Samples were run in triplicate, and at least 3 independent embryos for each genotype were analyzed. The relative expression of the various mRNAs was normalized to the expression of glyceraldehyde 3-phosphate dehydrogenase. The following primers were used. *Gli1*: forward 5′-ATCACCTGTTGGGGATGCTGGAT-3′, reverse 5′-GGCGTGAATAGGACTTCCGACAG-3′ *Gli2*: forward 5′-GTTCCAAGGCCTACTCTCGCCTG-3′, reverse 5′-CTTGAGCAGTGGAGCACGGACAT-3′ *Gli3*: forward 5′-AGCAACCAGGAGCCTGAAGTCAT-3′, reverse 5′-GTCTTGAGTAGGCTTTTGTGCAA-3′. *GAPDH*: forward 5′-TGC AGT GGC AAA GTG GAG AT-3′, reverse 5′-TTT GCC GTG AGT GGA GTC ATA-3′.

### Scanning Electron Microscopy

Freshly removed embryos were fixed with 2% PFA and 2% glutaraldehyde in 0.1 M sodium cacodylate buffer. Embryos were dissected to expose the lumen of the neural tube in 0.1M cacodylate buffer and post-fixed in osmium tetroxide for 1 hour. Embryos were then dehydrated in an ethanol series, dried with a Pelco Critical Point Drying Apparatus, mounted on aluminum stubs and sputter coated with 10 nm gold/palladium and viewed using a FEI Quanta 200 Scanning Electron Microscope. All images were acquired digitally.

### Transmission Electron Microscopy

Freshly removed embryos were immersed overnight in 0.1 M Sorensen’s phosphate buffer containing 2% glutaraldehyde and 2% paraformaldehyde, washed in 0.1 M Sorensen’s phosphate buffer, post-fixed in 1% OsO_4_ aqueous solution, washed in distilled water, dehydrated in a series of acetone and infiltrated using Polybed 812 epoxy resin. Blocks were polymerized at 60 °C. 60–90 nm ultrathin sagittal cross-sections were placed on uncoated 200 mesh copper grids, stained with 2% uranyl acetate aqueous and Reynold’s lead citrate and examined by scanning under a JEOL 1230 transmission electron microscope. Digital images were acquired using a KeenView high-resolution camera and Soft Imaging Solutions AnalySIS ITEM digital software.

### GST-pulldown assays

For GST-pulldown assays, GST-EHD1 (20 μg) was incubated with 40 μL glutathione Sepharose-4B beads (30 °C; 1 hour) followed by incubation with cell extracts (4 °C; overnight), and bound proteins were analyzed by Western blotting.

### Co-Immunoprecipitation Assays

SMO was co-immunoprecipitated from whole cell lysates using an EHD1 antibody (Abcam #ab109311) covalently bound (via cross-linker) to Protein G magnetic beads (Dynabeads, Invitrogen #10003D). SMO was detected by immunoblotting with an antibody specific for Smoothened (#ab38686)

### Data Analysis

#### Microscopy

For quantitative analyses of fluorescence intensities, all images were obtained with an identical gain, offset, and laser power settings. Data were imported into GraphPad Prism for graphing and statistical analysis using one-way ANOVA with a Tukey post-test analysis. Mean differences between groups were analyzed using Student’s t-test (two-tailed) or by ANOVA for more than two groups. P < 0.05 was considered statistically significant.

#### Western blots

Films were scanned with a photo scanner as grayscale TIFF files. Quantitative analysis of band intensities was performed with ImageJ using the gel lane tool and the data were transferred to GraphPad Prism for analysis.

## Results

### EHD1 deletion in a predominantly B6 background leads to mid-gestational embryonic lethality with major developmental defects

Previously, we established the *Ehd1*-null mice on a 129SV/B6 mixed background using EIIa-Cre mediated deletion of floxed *Ehd1* allele, and found that intercrossing of fully viable heterozygous mice yielded null pups at a sub-Mendelian ratio^30^, which was in contrast to another *Ehd1*-null mouse model on a 129Sv/Ev background^29^, in which no overt developmental or other phenotypes were observed. We bred the *Ehd1*-null allele further into B6 background for 5 generations and determined these mice to be 98% B6 by whole genome strain-specific marker analysis (Supplementary Fig. 1). Heterozygote crosses in this background yielded only WT and heterozygous pups and no *Ehd1*-null pups were observed in multiple independent mating. To assess if the lack of *Ehd1*-null pups in heterozygote mating was due to embryonic lethality and to determine the stage at which *Ehd1*-null embryos die, a genotype analyses was performed at various gestational stages on embryos arising from crossings of *Ehd1* heterozygous mice. Mating was tightly controlled and embryos were staged by somite counting of wild-type littermates. PCR-based embryo genotypes were assessed using the yolk sac membranes. While *Ehd1*-null embryos were present at or near the expected Mendelian ratios at E9.5 and E10.5, no *Ehd1*-null embryos were found at E11.5. (Table 1). Many of the *Ehd1*-null embryos showed signs of resorption at 10.5. These results put the stage of death of *Ehd1*-null embryos between E10.5 and E11.5, or approximately E11.

**Figure 1:**
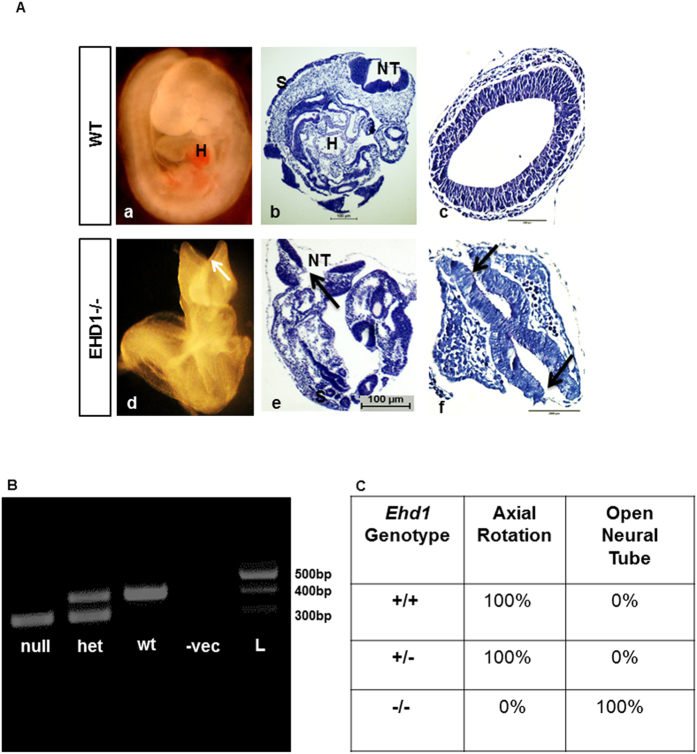
Major developmental defects in *Ehd1*-null embryos. (**A**) Whole mount (a & d); sagittal (b & e) and transverse (c & f) sections, of E9.5 WT control and *Ehd1*-null embryos are presented. Compared to the WT littermates, the *Ehd1-null* embryos are smaller, have fewer somites, displays neural-tube closure defects (arrows) and do not undergo axis rotation in the sagittal plane. Abbreviations: H, heart ventricle; S, somites; NT, neural tube. Scale bar, 100 μm. (**B**) PCR based genotyping of the *Ehd1* allele using embryonic yolk sac membranes. Wild-type band runs at 400 bp and the null band runs at 300 bp. (**C**) Table showing the phenotypic penetrance of the *Ehd1*–/– allele.

Gross examination of embryos at E9.5 revealed major developmental defects in *Ehd1*-null embryos (Fig. 1A) but no obvious defects in their placental development (data not shown). *Ehd1*-null embryos were smaller, displayed an open neural tube and showed failure of axial rotation. However, *Ehd1*-null embryos did not display left-right patterning defects. *Ehd1*-null embryos also displayed fewer somites than littermate controls (12–18 vs. 21–25 for control embryos), indicating impaired somitogenesis (Fig. 1A). *Ehd1*-null embryos also displayed delayed heart development; while all E9.5 *Ehd1*-null embryos exhibited a beating heart, the heart chambers were not developed as well as in controls (data not shown). Staining for endothelial cell marker PECAM1 did not show defects in vascularization in E9.5 *Ehd1*-null embryos (Supplementary Fig. 3). Staining for proliferating cells using phospho-Histone 3 (p-H3) revealed no significant changes in the proportion of proliferating cells in the neuroepithelium lining the open neural tubes in *Ehd1*-null embryos as compared to the controls (Supplementary Fig. 2A). No differences in the levels of apoptosis, measured by staining for cleaved caspase 3 (CC3) was observed between *Ehd1*-null and control embryos (Supplementary Fig. 2B). Collectively, these analyses demonstrated that *Ehd1* deficiency on a predominantly B6 background is embryonic lethal around day E11, and major defects in neural and cardiac development, axial rotation and somitogenesis precede the death of embryos.

### Expression of EHD family proteins during embryonic development

**Figure 2:**
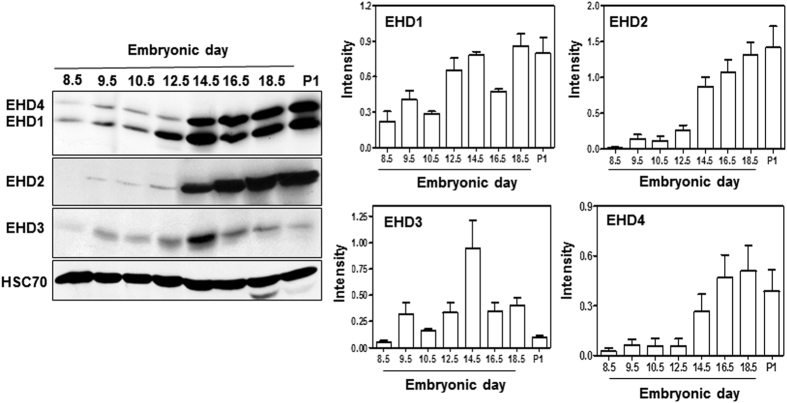
EHD protein expression during mouse embryonic development. Expression of EHD1, 2, 3 and 4 during embryonic development as revealed by western blotting of whole embryo lysates at the designated embryonic time points (E) and from total post-natal (P) fetal lysates of wild type mice. The EHD1/EHD4 membrane was serially stripped and re-probed with EHD2 antibody and a separate membrane was probed for EHD3. HSC 70 is the loading control. A single antibody recognizes both EHD1 and EHD4. The blot is a representative one from three individual experiments. Data from multiple experiments are presented as mean ± S.E.M. (error bars, n = 3) with levels of expression normalized to HSC70 expression in each experiment. Full-length blots/gels are presented in Supplementary Figure S7.

In view of the mid-gestational lethality of *Ehd1*-null embryos, we examined the expression of all four EHD proteins during early embryonic development, using western blotting of pooled embryo lysates with specific antibodies^27^. EHD1, EHD3 and EHD4 were detectable at E8.5 (the earliest time point examined), whereas the expression of EHD2 was first detectable at E9.5 (Fig. 2). EHD1 levels progressively increased to peak at E14.5 and remained high through the remainder of gestation into P1. EHD2 levels started to increase sharply at day 14.5 and continued to increase further through gestation. EHD3 expression showed a bell-shaped curve, peaking at E14, and sharply declining thereafter. EHD4 expression showed a pattern similar to that of EHD1. These findings indicate that the EHD family of protein expression begins early during embryogenesis and multiple members are expressed as early as E8.5, with substantial developmentally-regulated expression changes during the second half of gestation. The early expression of EHD1 is consistent with a role in embryogenesis, as revealed by phenotypic studies mentioned above.

**Figure 3:**
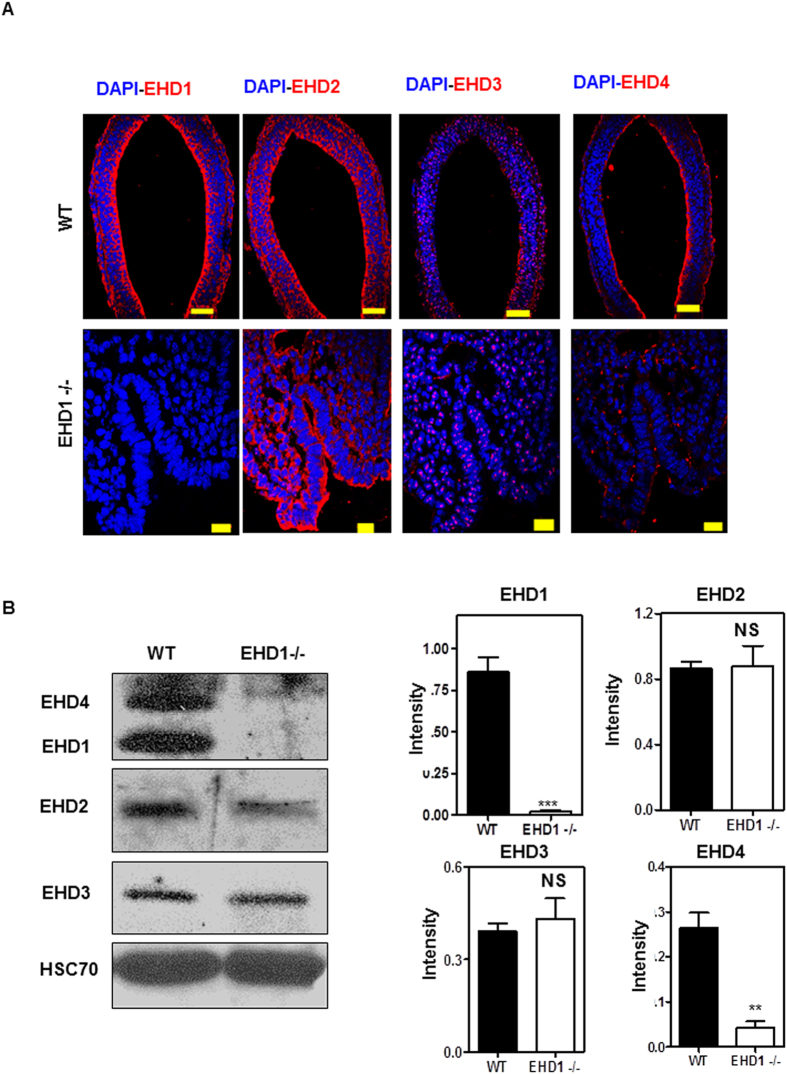
Expression and localization of EHD proteins in WT and *Ehd1*-null embryos. (**A**) Immunofluorescence analysis was carried out to determine EHD family protein localization in formalin-fixed neural tube sections from E9.5 WT and *Ehd1*-null mice. EHD, red; nuclei are counter-stained with DAPI (blue). Scale Bar, 50 μm (WT) or 20 μm (*Ehd1*-null). (**B**) 40 μg aliquots of pooled E9.5 WT and *Ehd1*-null whole embryo lysate protein were separated using 8% SDS-PAGE and immunoblotted using rabbit antibodies against EHD1, EHD2, EHD3 and EHD4. HSC-70 is the loading control. The blot is a representative one from three individual experiments. Data from multiple experiments are presented as mean ± S.E. (error bars, n = 3) with levels of expression normalized to HSC70 expression in each experiment. The EHD1/EHD4 membrane was serially stripped and re-probed with EHD2 antibody and a separate membrane was probed for EHD3. A single antibody recognizes both EHD1 and EHD4. HSC 70 is the loading control.EHD4 levels are reduced (P < 0.05) in *Ehd1*-null embryos whereas the expression of EHD2 and EHD3 is unchanged. Unpaired t test; n = 3 for each condition. Full-length blots/gels are presented in Supplementary Figure S8.

Previous studies have demonstrated that genetic deletion of individual *Ehd* genes is often associated with compensatory upregulation in the expression of family members in a tissue-specific manner. Such compensatory changes have been observed upon deletion of *Ehd1* on a mixed 129/B6 genetic background^30^,^32^ or of *Ehd4*^33^. Given the dramatically more penetrant phenotype of *Ehd1* deletion on a predominantly B6 background, as shown above, we examined the expression of all EHD proteins by western blotting of day E9.5 embryo lysates. Surprisingly, a significant reduction (p = 0.0038) in the level of EHD4 protein was seen in *Ehd1-null* compared to control embryo lysates, while the levels of EHD3 and EHD2 were comparable between *Ehd1*-null and control embryo lysates (Fig. 3B).

To further confirm the changes in the expression EHD family members and to assess the subcellular localization of EHD1, immunofluorescence staining of E9.5 embryos was carried out using previously-established antibodies^27^, with a focus on staining in the neural tube region as its development is drastically affected in the *Ehd1*-null embryos. In control embryos, EHD1, EHD2 and EHD4 are localized throughout the cytoplasm of neuroepithelial cells with prominent enrichment underneath the apical surface (facing the lumen), especially for EHD1 and 4 (Fig. 3A). EHD2 also displayed a similar pattern in the neural tube. In contrast, EHD3 shows a distinct, perinuclear localization in both the neuroepithelial and the cranial mesenchyme cells (Fig. 3A). These studies show that EHD family members are prominently expressed in the neural tube of developing embryos, and EHD1 and 4, and to a certain extent EHD2, show polarized distribution underneath the apical membrane of polarized neuroepithelial cells.

As anticipated, EHD1 staining was completely absent in E9.5 *Ehd1*-null embryonic neural tubes (Fig. 3A). Consistent with western blotting results, the intensity of EHD4 staining was markedly reduced in *Ehd1*-null embryos (Fig. 3A), although the subapical localization of the remaining signals remained unchanged. As noticed in Western blots, the intensity of EHD2 and EHD3 staining remained unchanged, and their localization was also comparable to the WT control embryos (Fig. 3A). The staining and blotting data together reveal that EHD protein expression is completely absent in *Ehd1*-null embryonic neural tubes, and for reasons that are not understood, the expression of EHD4 in the neural tube is markedly reduced. One of the proposed mechanisms for dosage-sensitivity is that proteins that participate in protein complexes are tuned to the fine balance of components (“balance hypothesis”) and overexpression/underexpression of one of the components can lead to mal-functioning of the whole complex^40^. Our results suggest that during neural tube development, EHD1 and EHD4 might together play some shared roles in cilia formation and SHH signaling though the exact mechanism still remains to be elucidated. In keeping with this notion, it has been shown that overexpressed EHD1 strongly interacts with EHD4 and a complex of endogenous EHD1 and EHD4 can be immuno-isolated from cells^41^.

In addition to EHD family members, we also assessed the expression of a number of proteins that have been implicated in EHD1 function in cellular studies. Western blot analyses of whole embryo lysates and whole cell lysates did not reveal any appreciable differences in the levels of Dynamin, Clathrin Heavy Chain, Adaptor Protein 2 (AP2), PACSIN2, Epsin-1, SNAP29, major endocytic proteins that are known to physically or functionally interact with EHD1^18^,^19^,^20^,^21^,^22^,^37^ (Supplementary Fig. 4 A and 4C). Immunofluorescence localization of these endocytic proteins was also carried out using embryonic neural tube sections as well as WT and *Ehd1*-null MEFS (Supplementary Fig. 4B). The expression or localization of these proteins in the neural tubes and MEFS was comparable between the WT and *Ehd1*-null E9.5 embryos or *Ehd1*-null MEFS. (Supplementary Fig. 4 A–D).

### Aberrant primary cilia morphology in the *Ehd1*-null neuroepithelium

As neural tube closure defect was a major structural abnormality in *Ehd1*-null embryos, we focused on potential mechanisms by which lack of EHD1 perturbs embryonic neural development. Primary cilia, non-motile microtubule-based membrane projections found on nearly every vertebrate cell, function as a hub of SHH signaling during neural tube development^42^,^43^. Prior studies have demonstrated that aberrations in the structural, trafficking and signaling components of primary cilia lead to developmental disorders, including neural tube closure defects^43^,^44^,^45^. The microtubule motors in primary cilia provide directional vesicular transport of proteins to and from the ciliary membrane, and direct links between vesicle trafficking and primary cilium formation and function have emerged recently from studies that have implicated endocytic proteins including members of the Rab and Arf/Arl subfamilies of the Ras superfamily of small GTPases, such as Rab8, Rab11, Rab23, Arl6, Arl13b in ciliary function^46^,^47^,^48^,^49^,^50^,^51^,^52^,^53^,^54^,^55^. As EHD1 is an endocytic recycling regulator functionally linked to Rab proteins^26^,^27^, we undertook an examination of the primary cilia structure on neuroepithelial cells lining the neural plate of E9.5 day embryos.

**Figure 4:**
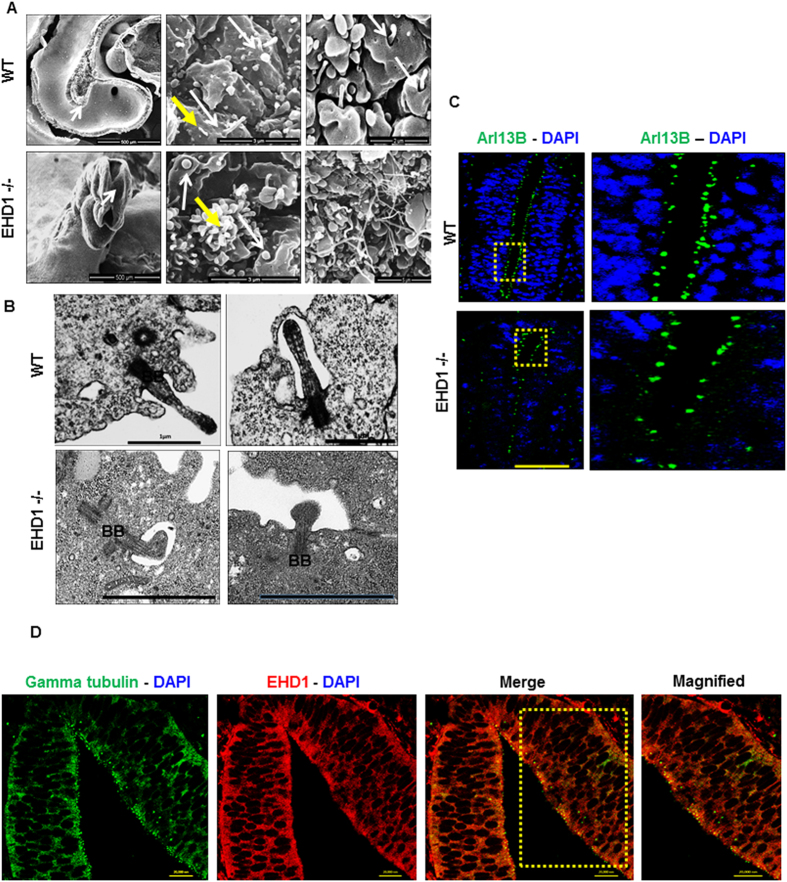
Altered primary cilia in *Ehd1*-null embryos. (**A**) Scanning electron micrographs whole-mount of WT and *Ehd1*-null embryos, with arrows in left panels showing the area scanned. White arrows are pointing to primary cilia emanating from ciliary pockets. Note the short and stubby cilia on the *Ehd1*-null neuroepithelium compared to the WT control. Right lower panel highlights the long and profuse cytoplasmic protrusions observed in the *Ehd1*-null neuroepithelium as compared with the WT neuroepithelium. Yellow arrows point to filopodia like cytoplasmic extensions. (**B**) Transmission Electron Microscopy (TEM) images showing stubby malformed primary cilia in the neuroepithelium of the *Ehd1*-null (lower panels) embryos as compared to their WT littermate controls (upper panels).Basal body (BB) structure is unchanged between WT control and *Ehd1*-null embryos. (**C**) Transverse sections of the neural tubes of E9.5 WT control and *Ehd1*-null embryos immune-stained for Arl-13B, a ciliary axonemal marker. The neuroepithelium of the *Ehd1*-null embryo shows sparse Arl-13b staining in comparison to the WT littermate control. Region of interest (box in left panels) has been magnified on the right. Scale Bar, 20 μm. (**D**) Transverse sections of the E9.5 WT control and *Ehd1*-null embryonic neural tubes were co-stained with antibodies against ciliary basal body marker gamma-tubulin and EHD1. EHD1 is seen in the same compartments of the neural tube as gamma-tubulin. Scale Bar, 20 μm.

We carried out scanning electron microscopy (SEM) studies, scanning the apical surface of neuroepithelial cells lining the floor of the neural plate at E9.5 (Fig. 4A). As expected, single primary cilia emerging from ciliary pockets were identifiable on each neuroepithelial cell in WT embryos (Fig. 4A). In addition, a large number of smaller, non-ciliary filopodia like cytoplasmic extensions were seen, as reported by others^56^. In contrast, neuroepithelial cells of *Ehd1*-null embryos exhibited substantially shorter stubby and bulbous ciliary structures projecting from apparently normal ciliary pockets (Fig. 4A). The filopodia like extensions on *Ehd1*-null neuroepithelial cells were much longer and profuse in comparison to those in control embryos. To confirm these results, transmission electron microscopy (TEM) studies were performed. WT neuroepitheliam displayed well-developed primary cilia projecting from ciliary pockets, with a well-defined basal body linking the ciliary membrane with the plasma membrane^57^. In contrast, the cilia on *Ehd1*-null neuroepithelial cells were smaller and barely projected out of the ciliary pocket (Fig. 4B). The positioning of basal bodies, which correspond to mother and daughter centrioles in the interphase located near the base of primary cilia, were comparable between WT and *Ehd1*-null neuroepithelial cells (Fig. 4B). Scanning electron micrographs were difficult to quantitate as the malformed cilia in the *Ehd1*-null neural tube were sparsely scattered and the numerous filopodia-like extensions made it extremely difficult to distinguish the actual cilia from the filopodia-like extensions. TEM images were quantitated from multiple images taken of random fields. This too proposed a challenge due to sparse cilia formation in the *Ehd1*-null neuroepithelium but we were able to distinguish primary cilia based on their axonemal architecture. To summarize the results, all primary cilia imaged for the EHD1 WT neuroepithelium at E9.5 looked normal and well-formed. However, out of the 30 cilia that we were able to image using TEM in the *Ehd1*-null embryos, only two resembled WT primary cilia.

Immunostaining for Arl13B, a specific marker of primary cilia^58^, showed punctate staining of the embryonic neuroepithelium in WT embryos (Fig. 4C). In contrast, Arl13B staining was substantially sparser in the *Ehd1-null* embryonic neuroepithelium (Fig. 4C). Co-staining for EHD1 revealed its localization in the same compartment as gamma tubulin, a specific marker of ciliary basal body on the luminal surface of neuroepithelial cells (Fig. 4D). Altogether, these studies demonstrate that EHD1 is required for normal cilia formation on neuroepithelial cells lining the developing neural tube and that EHD1 is in the same compartment as gamma tubulin in the developing neural tube.

### Developing neural tubes of *Ehd1*-null embryos exhibit evidence of aberrant SHH signaling

**Figure 5:**
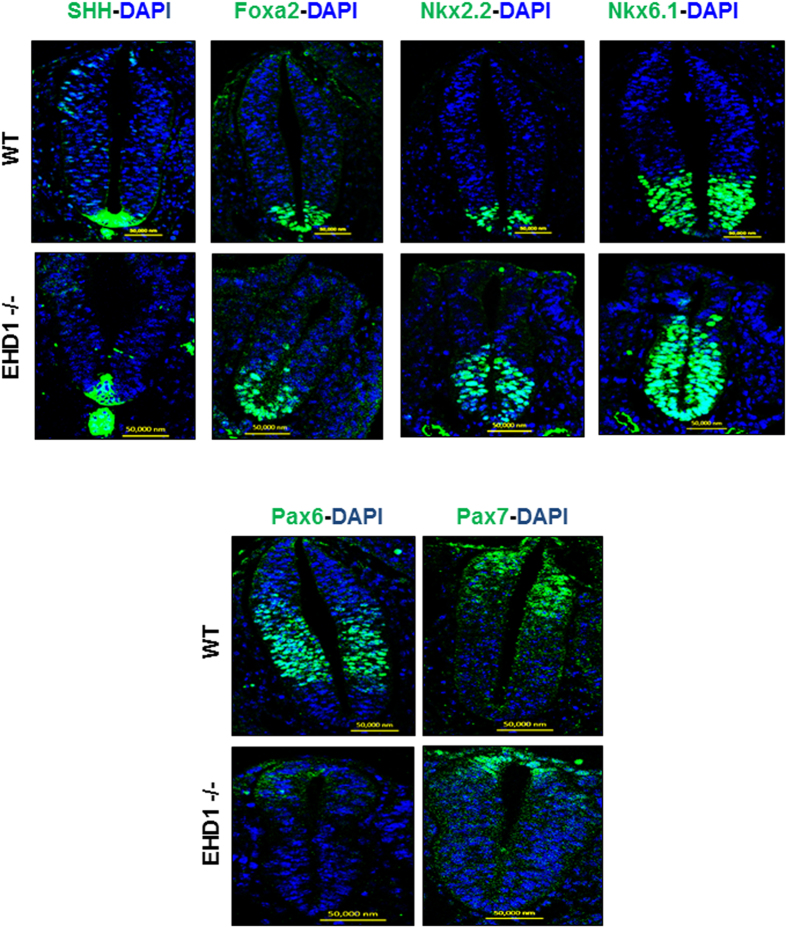
Neural tube patterning in *Ehd1*-null embryos suggest hyper-active SHH signaling. Transverse sections of the E9.5 WT control and *Ehd1*-null embryonic neural tubes (at the branchial level) were stained with antibodies against markers of dorsal (Pax6, Pax7) or ventral (Nkx2.2, Foxa2 and Nkx6.1) neuronal domains together with DAPI (nuclei). Note reduced dorsal and expanded ventral domains in *Ehd1*-null neural tubes; SHH staining was unaltered Scale Bar, 50 μm. Note that in the *Ehd1*-null embryo sections at the branchial level, the neural tube was closed from a point opposite the outflow tract to the proximal part of the tail. For clarification see supplementary Fig. S13.

Given a primary cilia defect on developing neuroepithelial cells of *Ehd1*-null mice, we assessed if SHH signaling and SHH-dependent dorso-lateral neural identity specification are altered in *Ehd1*-null mice. SHH is secreted by cells lining the base of neural fold and disseminates dorsally to create a morphogen gradient, which together with other morphogens, such as nodal and BMPs, controls the ventral, intermediate and dorsal domain specification during neural tube development, and aberrant SHH signaling is commonly associated with defective specification of dorsal and ventral neural identities^59^,^60^,^61^,^62^,^63^. The ventral, intermediate and dorsal identities can be distinguished by staining for specific transcription factors such as Foxa2 and Nkx6.1 for ventral domain and Pax6 and Pax7 for dorsal domains as seen for WT embryos (Fig. 5). In contrast to WT neural tubes, developing *Ehd1-null* neural tubes exhibited severe reduction in staining for markers of dorsal cell identities (Pax6 & Pax7) and an increased staining for markers of ventral identities (Foxa2 and Nkx6.1), consistent with an increase in SHH signaling (Fig. 5). Staining for SHH itself was comparable between the WT and *Ehd1*-null neural tubes (Fig. 5).

**Figure 6:**
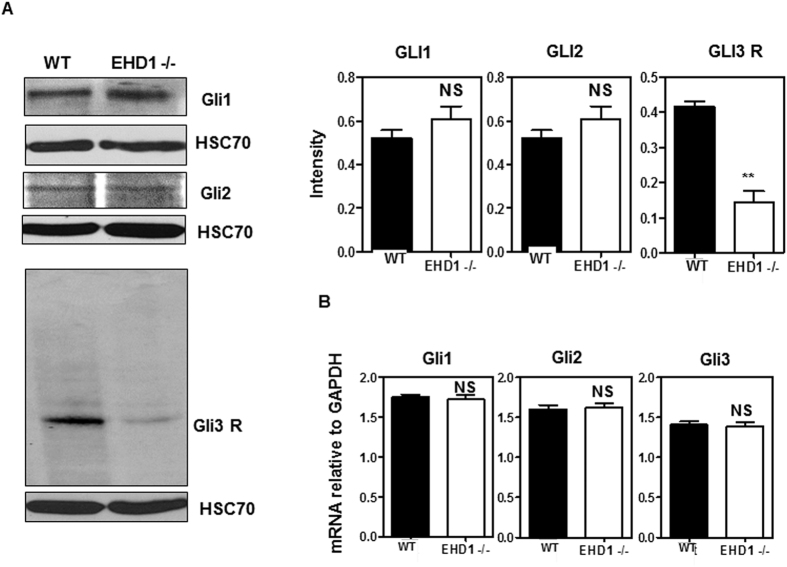
*Ehd1*-null embryos reveal features indicative of increased SHH signaling. (**A**) 40 μg aliquots of whole embryo lysate protein from pooled E9.5 WT and *Ehd1*-null embryos were separated using 8% SDS-PAGE and immunoblotted using antibodies against GLI1, GLI2 and GLI3. HSC-70 is the loading control. The blot is a representative one from three individual experiments. Data from multiple experiments are presented as mean ± S.E.M. (error bars, n = 3) with levels of expression normalized to HSC70 expression in each experiment. The membrane for GLI1 was serially stripped and reprobed with GLI2, followed by Hsc70 antibodies. A separate membrane was probed for GLI3.GLI3 repressor levels are markedly reduced in *Ehd1*-null embryos (P < 0.05) whereas GLI1 and GLI2 expression are unchanged between *Ehd1*-null and WT control littermates. Unpaired t test; n = 3 for each condition. Full-length blots/gels are presented in Supplementary Figure S9. (**B**) Relative mRNA levels of Gli1, Gli2 and Gli3 in the *Ehd1*-null and WT embryos measured by qRT-PCR analysis. Gli1, Gli2 and Gli3 mRNA levels remain comparable between *Ehd1*-null and WT embryos. Unpaired t test; n = 3 for each condition.

SHH promotes the internalization and degradation of its receptor Patched to remove its repressive effect on SHH signaling, and increases the trafficking of Smoothened into primary cilia where it signals to turn on the expression of activating GLI transcription factors, GLI1/2, and reduces the levels of GLI3 repressor^64^. Western blotting of whole embryo lysates revealed significantly reduced levels of GLI3 repressor protein in *Ehd1*-null embryos (p = 0.0002) compared to WT embryos whereas the levels of GLI1 and GLI2 were comparable (Fig. 6A). Reduction in GLI3 repressor levels in *Ehd1*-null embryos is also indicative of enhanced SHH signaling. We further used qPCR to assess the transcript levels of GLI transcription factors in WT vs. *Ehd1*-null embryos. Notably, changes in Gli1, Gli2 and Gli3 mRNA levels remained non-significant between WT and *Ehd1*-null embryos implying that depletion of EHD1 affects GLI3 repressor stability at the protein level (Fig. 6B).

### EHD1 regulates primary cilia morphogenesis in Mouse Embryonic Fibroblast cells

**Figure 7:**
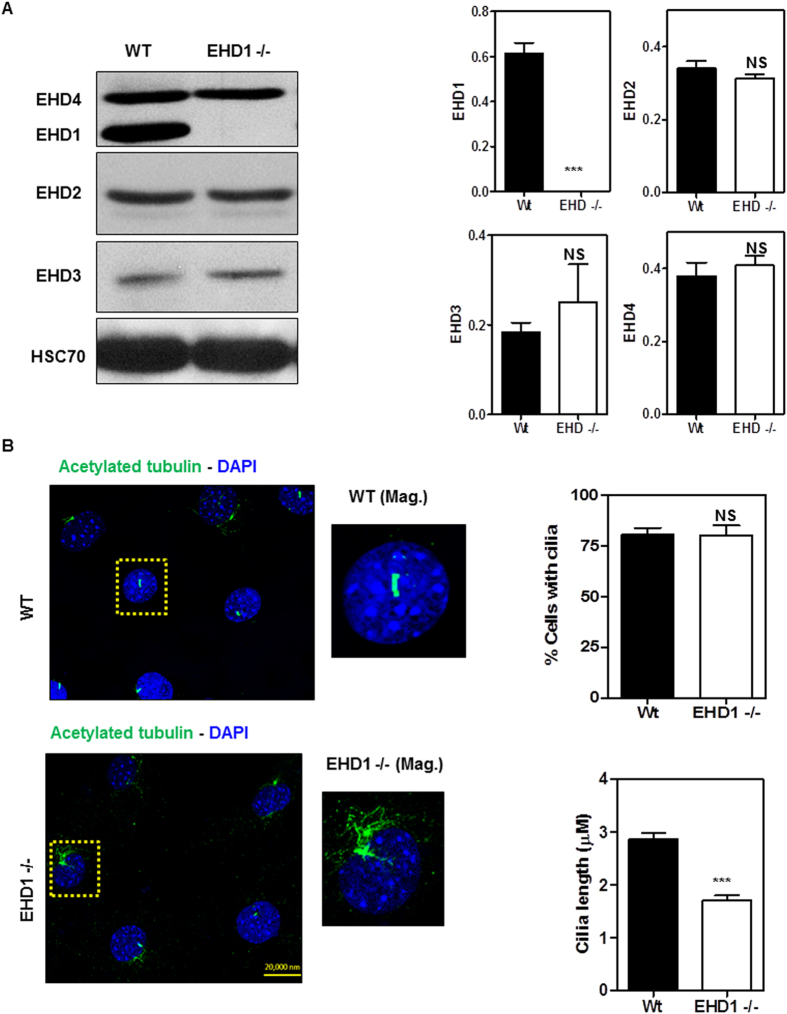
EHD1 regulates primary cilia morphogenesis in MEFS. (**A**) EHD1-null MEFS were derived from EHD1 floxed/floxed MEFS using Adenoviral mediated deletion and EHD1 deletion was confirmed using a western blot. 40 μg of protein from E9.5 WT and EHD1-null MEF lysates were separated using 8% SDS-PAGE and immunoblotting was done using antibodies raised against EHD1, EHD2, EHD3 and EHD4.HSC-70 is the loading control. Data from multiple experiments are presented as mean ± S.E. (error bars, n = 3) with levels of expression normalized to HSC70 expression in each experiment. Deletion of EHD1 does not lead to a significant change in overall expression levels of EHD2, EHD3 and EHD4. Full-length blots/gels are presented in Supplementary Figure S10. (**B**) WT control and EHD1-null MEFS were immunostained for acetylated tubulin, a primary cilia axonemal marker. DAPI stains the nucleus. The length of the cilia are significantly reduced in EHD1-null MEFS. (P < 0.0001) (Unpaired t test; n = 3 for each condition). EHD1-null MEFS show no significant change in the total number of ciliated cells.

To investigate if the primary cilia and SHH signaling defects observed in EHD1-null neural tubes could be replicated in a heterologous cell system, we prepared immortal MEFs from Ehd1^flox/flox^ mice and then infected these with adenoviruses expressing GFP (control) or Cre/GFP, and respective FACS-isolated GFP + lines were used as an isogenic cell system to explore the impact of EHD1 deletion on ciliogenesis. Western blotting of cell lysates confirmed the complete lack of expression of EHD1 in *Ehd1*-null MEFs, but all other EHD family members were still expressed (Fig. 7A). Notably, we did not observe a reduction in the expression levels of EHD4 in *Ehd1*-null MEFS.

Extensive studies have demonstrated that primary cilia are induced when fibroblasts are deprived of growth factors to promote cell cycle withdrawal. As expected, most cells in culture developed single primary cilia, identifiable by staining for acetylated tubulin in control MEFs, when grown for 48 hours in low serum medium (Fig. 7B). Ciliary length (measured from base to tip) was significantly reduced (p ≤ 0.0001; Mean ± SEM 1.710 ± 0.09775 N = 51) in *Ehd1*-null MEFs compared to the length of cilia in WT cells (Mean ± SEM 2.872 ± 0.1121 N = 39; Fig. 7B).

### EHD1 localizes to the primary cilia and this localization is enhanced upon SHH signaling activation

**Figure 8:**
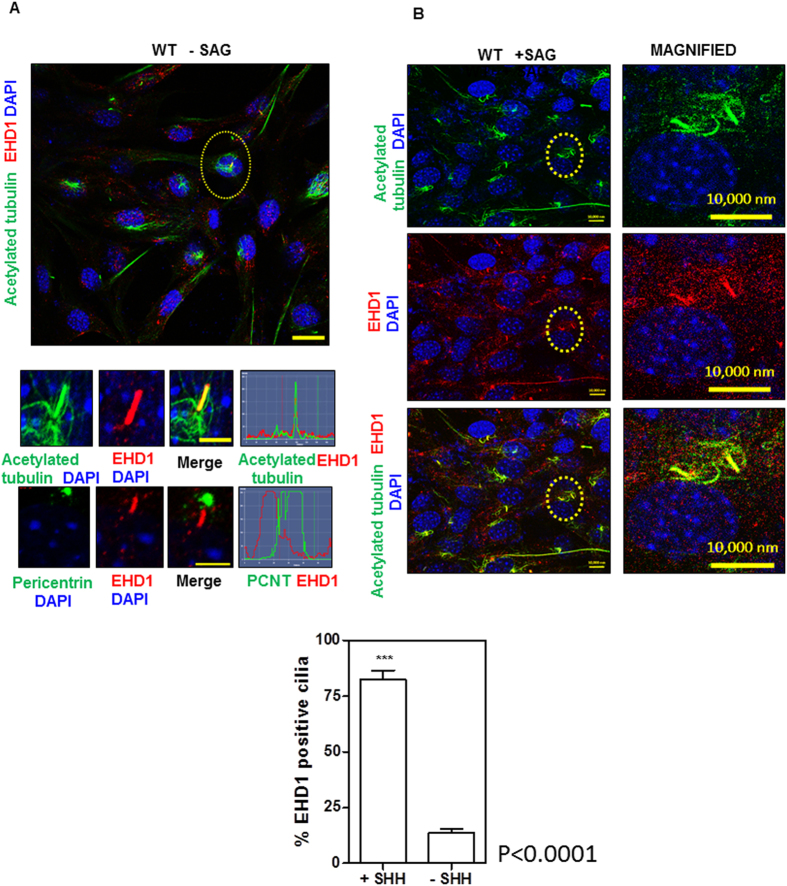
SHH pathway activation enhances EHD1 localization to the cilia. (**A**) WT MEFS were starved in low serum media for 48 hrs. before fixing and immunostaining for EHD1 and acetylated tubulin (ciliary axonemal marker) or EHD1 and Pericentrin (a basal body marker). Under conditions of serum starvation and no SHH pathway activation, EHD1 was found to co-localize with ciliary shaft marker acetylated tubulin in only 5% of cells studied. A profile scan for the ciliary shaft in the merged panel is shown on the right. EHD1 did not co-localize with the basal body marker Pericentrin. (**B**) WT MEFS were starved in low serum media for 24 hrs. and then treated with SAG in low serum media for another 24 hrs. to activate the SHH pathway before fixing and immunostaining for EHD1 and acetylated tubulin (ciliary axonemal marker). Under these conditions of SHH pathway activation, EHD1 localization to the cilia was significantly enhanced (P < 0.0001) and EHD1 was found to co-localize with ciliary shaft marker acetylated tubulin in 90% of cells studied thus leading us to conclude that EHD1 plays an essential role in regulating the SHH signaling pathway most probably by trafficking some essential SHH signaling component to the primary cilia.

To define if EHD1 localizes to the primary cilia itself, we used immunofluorescence to co-stain WT MEFS that had been serum-starved for 48 h for acetylated tubulin and EHD1. Under these conditions of serum starvation but without SHH pathway activation, EHD1 was visible along the entire length of the cilium but this localization was a rare event and found in only 5% of the cells counted. EHD1 did not co-localize with the basal body marker pericentrin (Fig. 8A). The regulation of protein delivery and movement within primary cilia is key to regulation of the ciliary SHH pathway. Almost all components of the Sonic hedgehog (Shh) signaling pathway are localized to the cilium, and their localization shifts in response to the SHH ligand^65^,^66^,^67^,^68^,^69^. To assess if EHD1 plays a role in regulating the SHH signaling pathway potentially by trafficking essential signaling components to the primary cilia, we co-stained WT MEFS that had been serum-starved for 24 h and then treated with SAG for another 24 h to activate the SHH pathway with acetylated tubulin and EHD1. Treatment with SAG lead to a dramatic and significant (p < 0.0001) enhancement of EHD1 localization to the cilia and immuno-staining revealed a co-localization of EHD1 in the primary cilia of 90% cells under conditions of SHH pathway activation thus leading us to conclude that EHD1 is likely to play a role in the traffic of some SHH signaling component to the primary cilia (Fig. 8B).

### EHD1 depletion leads to aberrant SHH signaling in MEFS

**Figure 9:**
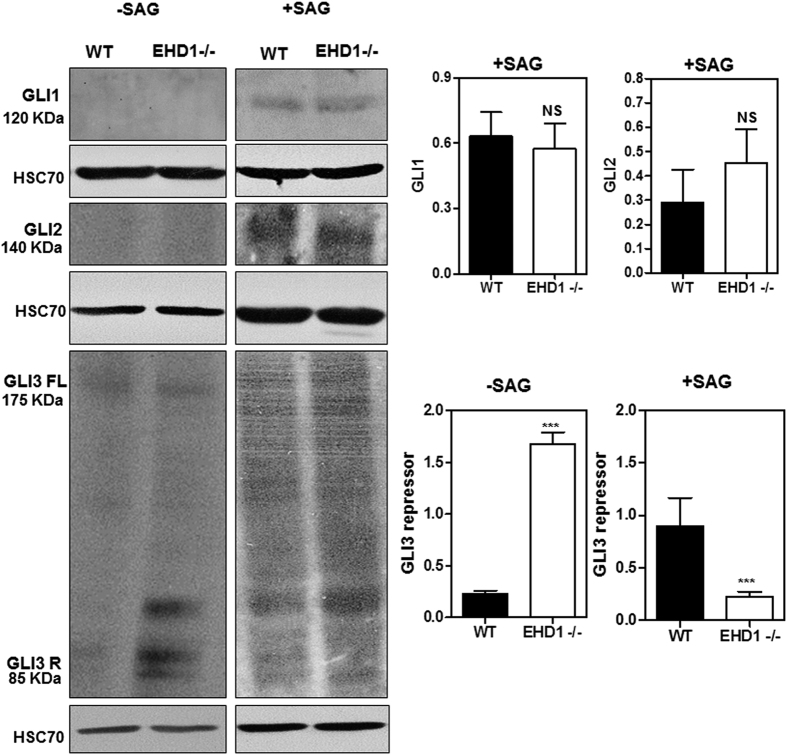
*Ehd1*-null MEFS reveal features indicative of increased SHH signaling. WT MEFS and EHD1-null MEFS were starved in low serum media for 24 hrs. and then either treated with SAG in low serum media for another 24 hrs. to activate the SHH pathway or left untreated in low serum media for another 24 hrs. 40 μg aliquots of whole cell lysate protein from pooled WT and *Ehd1*-null MEFS under both these conditions were separated using 8% SDS-PAGE and immunoblotted using antibodies against GLI1, GLI2 and GLI3. HSC-70 is the loading control. The blot is a representative one from three individual experiments. Data from multiple experiments are presented as mean ± S.E.M. (error bars, n = 3) with levels of expression normalized to HSC70 expression in each experiment. GLI3 repressor levels are significantly reduced in *Ehd1*-null MEF lysates (P < 0.05) treated with SAG.GLI1 and GLI2 expression are unchanged between *Ehd1*-null and WT MEF lysates with or without SHH pathway activation. Unpaired t test; n = 3 for each condition. Full-length blots/gels are presented in Supplementary Figure S11. We also observed that under unstimulated conditions, levels of the GLI3R were significantly elevated (p < 0.0002) in the *Ehd1*-null MEFS presumably to prevent the hyper activation of the pathway in the absence of ligand. Levels of GLI1 and GLI2 remained comparable between WT and *Ehd1*-null MEFS under stimulated and unstimulated conditions (Fig. 9).

As EHD1 localization to the cilia in WT MEFS drastically increases upon SHH pathway activation and EHD1-null mouse embryos show SHH pathway hyper-activation, we hypothesized that EHD1 functions in the cilium to regulate SHH signaling. Since the goal of SHH signaling is to control the balance of GliA and GliR, and this balance requires cilia, we investigated SHH signaling status in WT Vs EHD1-null MEFS with and without SHH stimulation. Endogenous GLIs were difficult to analyze using immunoblotting but importantly, we found a significant reduction (p < 0.05) in the expression levels of the GLI3 repressor in the EHD1-null MEFS upon SHH pathway stimulation with SAG indicating that even in EHD1-null MEFs there is a hyper activation of the SHH signaling pathway (Fig. 9). We also observed that under unstimulated conditions, the levels of the GLI3R were significantly elevated (p = 0.0002) in the EHD1-null MEFS presumably to prevent the hyper activation of the pathway in the absence of ligand. Levels of GLI1 and GLI2 remained comparable between WT and EHD1-null MEFS under stimulated and unstimulated conditions (Fig. 9).

### The localization of SHH signaling component SMO is disrupted in EHD1-null MEFS and EHD1 co-localizes with SMO upon SHH pathway activation

**Figure 10:**
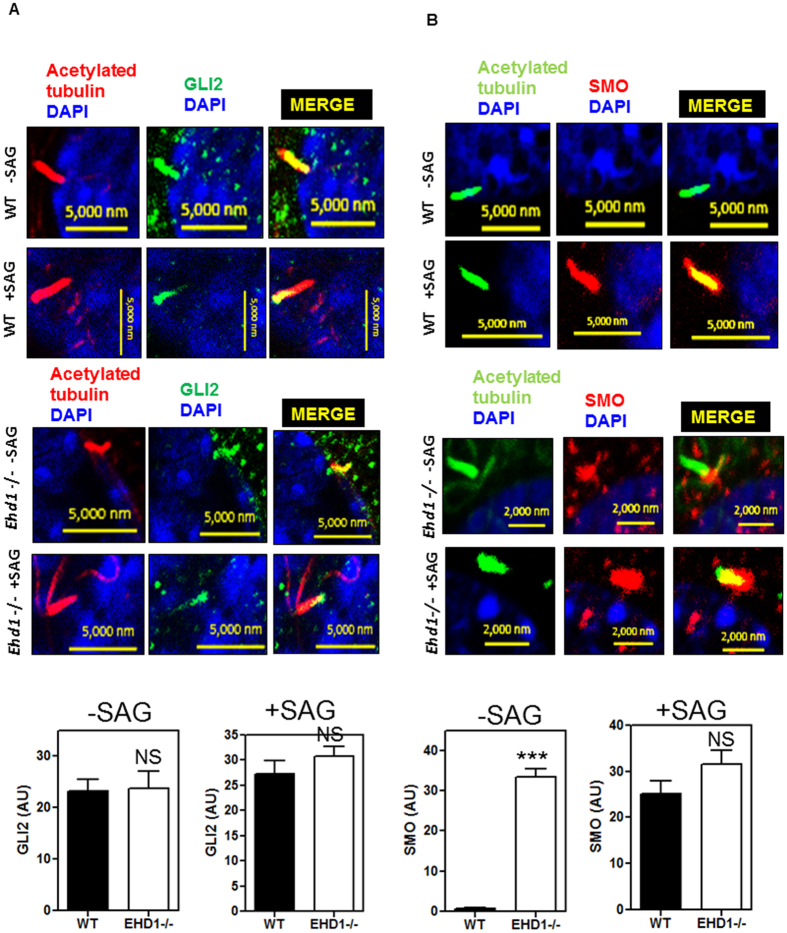
The dynamic localization of the SHH signaling component SMO is disrupted in EHD1-null MEFS. WT MEFS and *Ehd1*-null MEFS were starved in low serum media for 24 hrs. and then either treated with SAG in low serum media for another 24 hrs. to activate the SHH pathway or left untreated in low serum media for another 24 hrs. before subjecting them to immunostaining with antibodies against acetylated tubulin and endogenous SHH pathway components including SMO and GLI2. (**A**) In both WT and EHD1-null MEFS GLI2 decorates the entire cilia without SHH pathway activation and upon pathway activation, GLI2 was found to be enriched at the ciliary tips in both EHD1 WT and *Ehd1*-null MEFS. (**B**) In WT MEFS SMO was found to translocate to the primary cilia only upon SHH pathway activation and under conditions of no stimulation, SMO was completely absent from the ciliary shaft. However, regulated traffic of SMO to the cilia in response to SHH pathway activation was completely abolished in *Ehd1*-null MEFS where SMO was found to be localized to the cilium without SHH stimulation. This important finding indicated to us that EHD1 plays a critical role in regulating the entry of SMO into cilia. SMO was further enriched on SHH stimulation in *Ehd1*-null MEFS.

**Figure 11:**
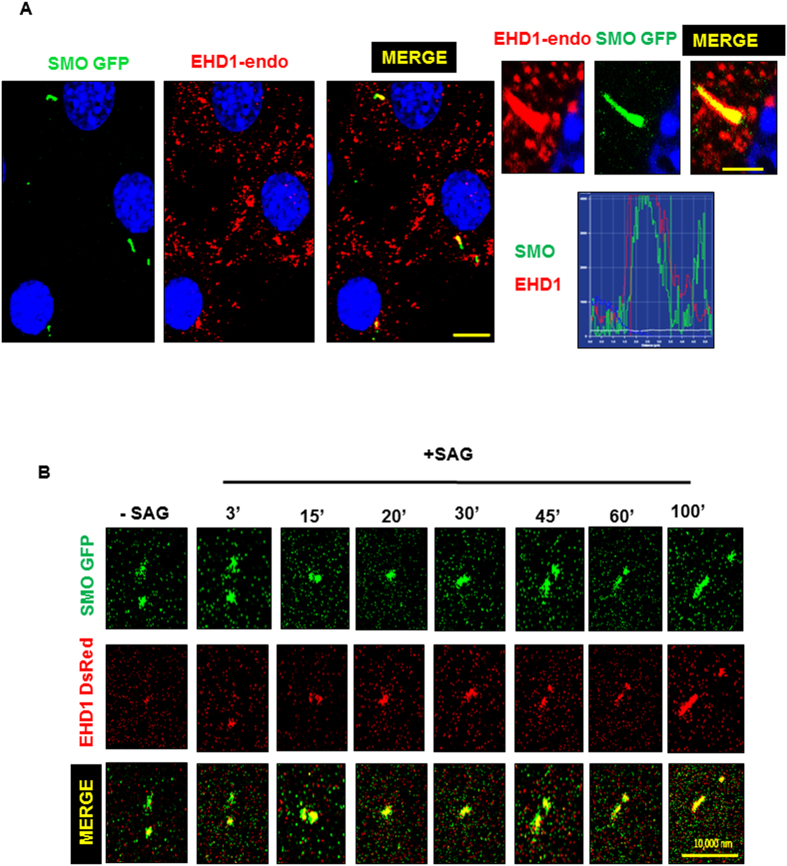
EHD1 co-localizes and co-traffics with SMO to the cilia upon SHH pathway stimulation. (**A**) WT NIH3T3 cells stably expressing SMO-GFP were starved in low serum media for 24 hours and stimulated with SAG in starvation media for another 24 hours. Under these conditions EHD1 was seen to co-localize with SMO in the primary cilia. A profile scan for SMO and EHD1 in the primary cilia merged panel is shown. Under these conditions, EHD1 was seen to traffic to the cilia of 60% cells studied. (**B**) WT NIH3T3 cells stably expressing SMO-GFP and transiently expressing EHD1-DsRed were starved in low serum media for 24 hours and stimulated with SAG immediately before starting live imaging of Smoothened and EHD1.As reported in earlier studies, Smoothened was found in preciliary vesicles under non-stimulated conditions but upon SHH pathway activations, EHD1 was seen to associate with Smoothened vesicles and co-traffic with SMO into the primary cilia.

To better determine how SHH signaling is regulated by EHD1, we examined the dynamics of SHH components using antibodies against the endogenous proteins. Normally, GLI2 localizes to the entire ciliary axoneme and is enriched at the ciliary tip after SHH stimulation^68^,^69^. Indeed, when we measured the fluorescence intensity in the tips of cilia relative to background staining using antibodies that recognize full-length GLI2, we found that GLI2 was enriched in wild-type MEFs after treatment with SAG-conditioned media (Fig. 10A). Interestingly, there was no significant change in GLI2 localization to the entire cilia under basal conditions and GLI2 enrichment at the ciliary tips after SHH pathway activation (Fig. 10A) in EHD1-null MEFS. SMO and PTCH1 localize along the length of the cilium in a complementary manner: PTCH1 in the absence of ligand and SMO upon pathway stimulation^65^,^67^. As expected, we saw SMO ciliary levels increase after SHH stimulation in wild-type MEF (Fig. 10B). We investigated how these dynamics changed in the absence of EHD1 and interestingly, we found SMO localized to the cilium in EHD1-null MEFS without SHH stimulation (Fig. 10B), indicating that EHD1 plays a critical role in regulating the entry of SMO into cilia in response to SHH pathway activation. We found SMO was further enriched on SHH stimulation in EHD1-null MEFS (Fig. 10B). Importantly we found EHD1 to co-localize with SMO in the WT MEF cilia after SHH pathway activation in 70% of cells counted (Fig. 11A). To summarize, these results demonstrate a fundamental defect in the trafficking of SHH signaling protein SMO in EHD1-null MEFs.

### SMO is a novel binding partner of EHD1 and EHD1 aids in ciliary trafficking of SMO upon SHH pathway stimulation

**Figure 12:**
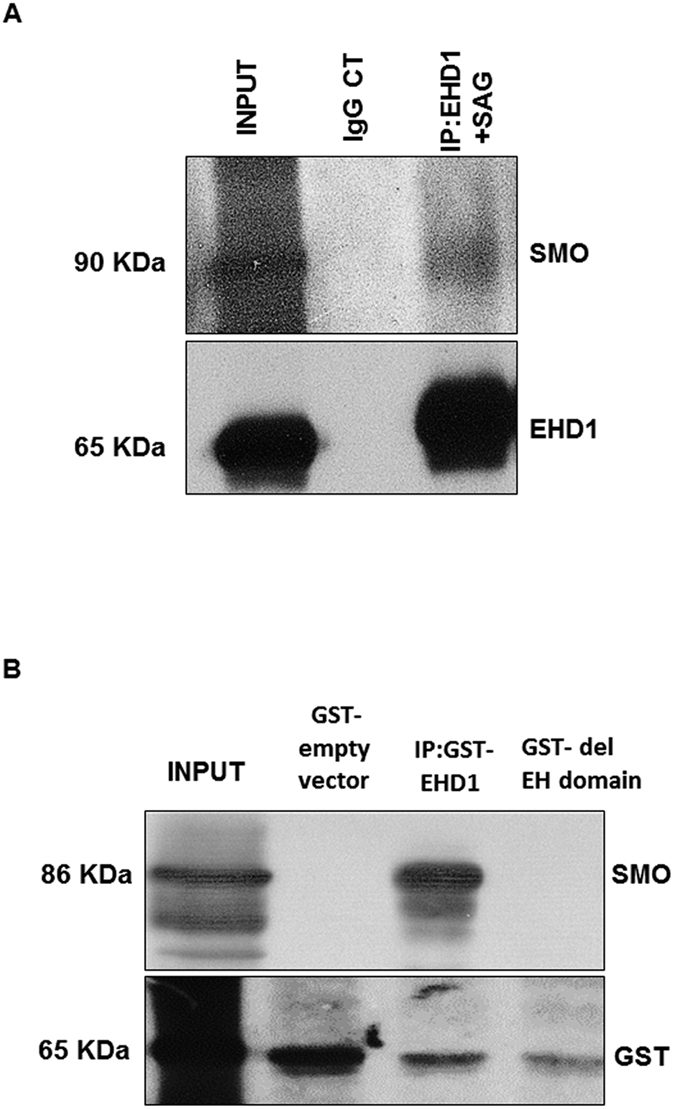
SMO is a novel binding partner of EHD1. (**A**) Immunoblotting was used to assess the presence of SMO in anti-EHD1 immunoprecipitates from WT NIH3T3 cells stably expressing SMO GFP treated with SAG (24 hrs). Input samples show the amount of each protein in the whole extract before the IP. Immunoblots showing the amount of SMO that co-precipitated with EHD1 from cells that stably expressed the protein and were treated with SAG for 24 hrs. A control IP was performed with non-specific rabbit IgG instead of EHD1 antibody and WT NIH3T3 cell lysates stably expressing SMO GFP treated with SAG (24 hrs). Arrows denote the molecular weight fraction of SMO that associates with EHD1. Full-length blots/gels are presented in Supplementary Figure S12A. (**B**) WT NIH3T3 cells stably expressing SMO-GFP were starved in low serum media for 24 hours and stimulated with SAG in starvation media for another 24 hours and lysed and these lysates were incubated with GST-EHD1 or GST-EH domain deleted EHD1. The membranes were probed with antibodies to Smoothened and GST.GST-fused to the empty plasmid vector was used as a negative control. The data is representative of three separate experimental repeats. Full-length blots/gels are presented in Supplementary Figure S12B.

A very recent study^70^ showed that EHD1 and Smoothened are co-localized to the same pre-ciliary vesicles upon conditions of serum starvation. Stimulated by our results demonstrating the mis-localization of SMO in the *Ehd1*-null cilia in absence of SHH ligand and the co-localization of SMO with EHD1 in response to SHH pathway activation, we set out to investigate if EHD1 aids in the trafficking of Smoothened into the primary cilia proper upon SHH pathway stimulation by SAG. We overexpressed DsRed EHD1 in NIH3T3 cells stably expressing SMO-GFP, serum starved these cells for 24 hours and added SAG (100 nM) to stimulate the SHH pathway immediately before live-cell imaging. Using live-cell imaging, we confirmed that EHD1 and Smoothened indeed co-localize at vesicular structures inside the cell-body under non-stimulated conditions; however, once SHH pathway is activated with the addition of SAG, EHD1 and Smoothened are co-trafficked into the primary cilia (Fig. 11B). This observation led us to consider whether EHD1 directly associates with SHH pathway component SMO. We tested this association using Immunoprecipitation assays with EHD1 antibody and WT NIH3T3 cell lysate stably expressing SMO GFP at endogenous levels that had been starved and stimulated with SAG. In this binding assay, SMO-GFP showed an interaction with EHD1. To demonstrate the specificity of the interaction, we included a control IP reaction where non-specific rabbit IgG was used to co-precipitate the target protein SMO instead of the specific EHD1 antibody (Fig. 12A). Spurred by the observation that SMO and EHD1 co-immuoprecipitate with each other, we further investigated the molecular basis of this interaction and whether this interaction is mediated by the EH domain of EHD1. This was addressed using *in vitro* GST-pulldown assay utilizing GST-EHD1 and NIH3T3 cells stably expressing SMO GFP. Incubation of GST-EHD1 with SMO-GFP lysates resulted in SMO pulldown (Fig. 12B), supporting a conclusion that SMO and EHD1 physically associate with each other. Notably, incubation of GST-EHD1 fusion protein lacking the EH domain with NIH3T3-SMO-GFP lysates failed to pull down SMO (Fig. 12B), consistent with the likelihood that EHD1-SMO interaction is mediated by the binding of the EH domain of EHD1 to a potential NPF motif present in the cytoplasmic tail of SMO.

## Discussion

Endocytic traffic of plasma membrane lipids and proteins provides a versatile mechanism to regulate cell-cell and cell-environment interactions across eukaryotes. The EHD family of proteins has recently emerged as a key player in the recycling arm of endocytic trafficking. The initial discovery of the single *C. elegans* EHD protein RME-1 hinted towards important physiological functions of EHD proteins in mammals but it is only recently that we and others have utilized mouse genetic models to explore the *in vivo* roles of EHD proteins. Here, we demonstrate that deletion of mouse *Ehd1*, on a predominantly B6 background, leads to embryonic lethality by mid-gestation due to developmental arrest at an early embryonic time point. A prominent manifestation of EHD1 deficiency was a neural tube closure defect associated with defective ciliogenesis, aberrantly increased SHH signaling and altered dorso-ventral neural identities. Our studies reveal a novel physiological role of EHD1-dependent endocytic recycling in the regulation of ciliogenesis and ciliary signaling, processes of fundamental importance in the development of neural tube and other organs.

As we have reported, genetic deletion of *Ehd1* on a mixed 129/B6 background was associated with substantial prenatal lethality^30^, while EHD1 deletion on a 129Sv/Ev background reported by another group was without any phenotype^29^, indicating a strong impact of the genetic background. Indeed, further backcross to a predominantly B6 background yielded complete embryonic lethality as we report here. It is well documented that the phenotypic effects of many individual mutant alleles are context dependent, with respect to environmental influences. Indeed, genetic background has long been known to influence observed phenotypic expression across organisms^71^,^72^,^73^,^74^,^75^,^76^,^77^,^78^,^79^. Although the basic influence of genetic background on the expressivity of mutations is well known, the consequences of such influences is poorly understood^80^. In recent years, modifier screens have been very important, and have helped in identifying large numbers of genes that interact to influence the visible expression of the phenotype of the focal mutation, even when the modifier may not have a visible phenotype by itself^74^,^81^. Such modifier screens will help answer the phenotypic variability of *Ehd1*-null mice depending on their genetic background.

We established the stage of death of the *Ehd1*-null embryos to be between E10.5-E11.5 (Table 1). Notably, *Ehd1*-null embryos displayed severe developmental defects prior to death, including open neural tubes, failure to undergo axial rotation, delayed somitogenesis and defective cardiac development (Fig. 1A). Mid-gestation lethality is often contributed by cardiac failure and vascular defects. While *Ehd1*-null embryos displayed delayed cardiac development (data not shown), vascular development in the embryo proper appeared to be normal based on PECAM1 staining (Supplementary Fig. 3). The combination of these defects together with ocular lens development defects in *Ehd1*-null mice on a B6/129 background (Arya P. *et al.* Manuscript Submitted) pointed to a partial similarity to ciliopathies, a group of genetic diseases in which defective primary cilia formation or function yields a plethora of developmental abnormalities, including the neural tube defects seen in *Ehd1*-null mice^44^,^45^,^82^. Furthermore, the overall combination of early embryonic developmental defects in *Ehd1*-null embryos on a predominantly B6 background was highly reminiscent of the embryonic phenotypes produced by the impairment of SHH signaling pathway, which has been implicated in somite formation^83^,^84^,^85^,^86^, cardiac development^87^,^88^,^89^,^90^, neural tube morphogenesis^91^,^92^,^93^ and axial rotation^94^,^95^. Recent studies have established that primary cilia are hubs for SHH signaling^96^. Together, our phenotypic examination of *Ehd1*-null mice and previous reports strongly supported a hypothesis that EHD1 was a regulator of ciliogenesis and SHH signaling. Studies described here provide evidence for both, establishing EHD1 as a novel player in regulating ciliogenesis and SHH signaling.

Scanning and transmission EM studies demonstrated short, stubby primary cilia on neuroepithelial cells lining the neural tube in *Ehd1*-null embryos (Fig. 4A,B). Cilia-specific marker staining showed a reduced number of ciliated neuroepithelial cells in these mice (Fig. 4C). Notably, EHD1 co-localized to the same compartment as the basal body marker gamma-tubulin on neuroepithelial cells (Fig. 4D). A key role of EHD1 in the formation of primary cilia was further confirmed in a heterologous system, using isogenic WT and EHD1-null MEFs. In these cells, the loss of EHD1 expression is associated with a dramatic decrease in cilia length (Fig. 7B). Thus, multiple lines of evidence suggested that altered primary cilia function may contribute to abnormal neural development in EHD1-null embryos. The short and stubby ciliary phenotype observed in the *Ehd1*-null embryos resembles that observed in models with loss of intra-flagellar transport protein complex, IFT-A, components^55^,^97^,^98^,^99^,^100^,^101^.

A key pathway that shapes the embryonic neural development and involves primary cilia is signaling by SHH^96^. As disruption of ciliary structure does not always affect SHH signaling, as shown by *Rfx3* mouse mutants, which have short cilia but normal SHH activity^102^, we wished to understand if in addition to regulating ciliary membrane biogenesis, EHD1 might regulate SHH signaling as well. SHH, secreted by the notochord and floor plate, forms a morphogen gradient from ventral to dorsal neural tube and, in conjunction with concentration gradients of other morphogens such as Nodal and BMPs, specifies neuronal cell fates^59^,^60^,^61^,^62^,^63^. In the neural tubes of EHD-null embryos at the cranial, branchial and lumbar arch levels, we observed significant expansion of the floor plate marker FOXA2 and of the V3 motor neuron and V2 interneuron marker NKX6.1 (Fig. 5). The expression pattern of V3 interneuron progenitor marker NKX2.2 also showed marked increase in the expression domain at the lumbar arch level and some NKX2.2 positive cells were found dispersed dorsally in *Ehd1*-null compared to the control embryos. A dramatic change in expression was also observed in the dorsal markers PAX6 and PAX7, with their expression domains shifted dorsally and restricted to a smaller domain than in wild-type embryos, suggesting a loss of dorsal neuronal domains in *Ehd1*-null embryos (Fig. 5). The dorso-ventral neural tube patterning defects seen in *Ehd1*-null embryos resemble the phenotypes associated with increase in SHH signaling. For example, the phenotypes we observed resemble those of mutants with functional disruption of negative regulators of SHH signaling, including Rab23, Gpr161, Thm1, Ptch1, TULP3 and PKA^97^,^103^,^104^,^105^,^106^,^107^,^108^,^109^,^110^. This similarity suggests that the primary function of EHD1 in the early embryo is to restrain the activity of the SHH pathway.

Potential mechanisms by which lack of EHD1 leads to increased SHH signaling in the developing neural tube are suggested by our analyses of WT vs. *Ehd1-null* embryos for aberrations in downstream components of SHH signaling pathway. The antibodies we tested failed to detect noticeable amounts of GLI3-190 in the pooled E9.5 WT lysates, possibly because full length GLI3 is readily processed, but blotting with antibodies against GLI3 protein identified predominantly the Gli3-83 repressor. We found severe down-regulation in the expression of GLI3 repressor, whose formation is known to be inhibited by SHH signaling^111^,^112^,^113^, in *Ehd1-null* embryos. There were however no significant differences in the expression levels of GLI1 and GLI2 between the *Ehd1*-null and control embryos (Fig. 6). While lack of an increase in GLI1 expression in *Ehd1-null* embryos is unexpected in the face of functional evidence of increased SHH signaling, our observations are supported by previous studies in which expression of full-length Gli3 protein was found to suppress the stimulatory effect of GLI1 and this suppressive effect was more dramatic with constructs encoding truncated GLI3 proteins^111^, which may dominantly suppress GLI1 function^111^. The high potency of truncated GLI3 proteins as suppressors of GLI-dependent transcription described in these studies suggests that the concentration of the endogenous GLI3-83 repressor is an important determinant of GLI-dependent SHH signal output. The ability of GLI3 and truncated GLI3 to suppress GLI1 transcription has been well characterized in several established cell lines including MNS70, NIH3T3, HK293 and C3H10T-1/2^114^,^115^,^116^,^117^. These studies help rationalize our findings and suggest that the pattern of GLI-dependent transcription within a developing structure is likely to depend on the balance of activities of all GLI protein species present. It is plausible that loss of EHD1 selectively controls the generation or stability of GLI3 repressor, and the unchanged GLI1/2 levels in the face of reduced GLI3 suppressor levels is perceived by developing neurons as an increase in SHH signaling. We also investigated the SHH signaling status in a heterologous system of WT Vs EHD1-null MEFS with and without SHH stimulation. Importantly, we found a similar significant reduction (p < 0.05) in the expression levels of the GLI3 repressor in the EHD1-null MEFS upon SHH pathway stimulation with SAG indicating that even in EHD1-null there is a hyper activation of the SHH signaling pathway (Fig. 9). We also observed that under unstimulated conditions, the levels of GLI3R were significantly (p = 0.0002) elevated in EHD1-null MEFS possibly to prevent the hyper activation of the pathway in the absence of the ligand (Fig. 9). The levels of GLI1 and GLI2 remained comparable between WT and EHD1-null MEFS under stimulated and unstimulated conditions (Fig. 9).

Regulation of protein delivery and movement within primary cilia is key to the control of ciliary SHH pathway. Almost all components of the Sonic hedgehog (SHH) signaling pathway are localized to the cilium, and their localization shifts in response to SHH ligand^65^,^66^,^67^,^68^,^69^. A role of EHD1 in regulating SHH signaling by controlling ciliary trafficking of certain essential SHH signaling components was suggested by co-staining studies in MEFs. EHD1 staining was visible along the entire length of the cilium in serum-starved MEFs without SHH pathway activation, but this localization was a rare event and found in only 5% of the cells counted. Under these conditions, EHD1 did not co-localize with the basal body marker pericentrin (Fig. 8A). Notably, immuno-staining revealed a co-localization of EHD1 in the primary cilia of 90% cells under conditions of SHH pathway activation thus strengthening our hypothesis that EHD1 plays a role in the trafficking of SHH signaling components to the primary cilia (Fig. 8B).

How might EHD1 regulate the complex process of ciliogenesis and SHH signaling? A recent study defined a role for EHD1 at a distinct step of ciliogenesis, in pre-ciliary vesicle formation during the very early stages of ciliogenesis, through interactions with Rab8/11 small GTPases^70^. This was not surprising as EHD1 regulates the Rab11 endosome recycling compartment trafficking and binds to Rab11-FIP2, a Rab11 effector^118^. Furthermore, EHD1 can bind to the Rab8 effector MICAL-L1 and aid in membrane tubulation and vesicle scission^11^,^19^. Our independent studies emanating from the phenotype of *Ehd1*-null mice and EHD1-null MEFS provide a complementary line of evidence to further support a key role of EHD1 in primary ciliogenesis. Although the defects in embryonic development in *Ehd1*-null mice may be an indirect effect of aberrant SHH signaling downstream of malformed primary cilia, it is also possible that the role of EHD1 to regulate SHH signaling is separate from its role to maintain cilia architecture, and it includes the endocytic traffic of signaling proteins into the cilia in response to SHH. As described below, our data suggest that EHD1 regulates the SHH signaling pathway at a step that is dependent on SMO ciliary localization.

To address the question of how EHD1 regulates ciliary morphogenesis and SHH signaling, we focused on the key role of EHD1 as an endocytic traffic regulator and asked if it could be trafficking ciliary proteins. Almost all components of the Sonic hedgehog (SHH) signaling pathway are localized to the cilium, and their localization shifts in response to the SHH ligand^65^,^66^,^67^,^68^,^69^. In the absence of ligand, the GLI transcription factors GLI2 and GLI3 are localized to the tips of cilia and are processed to form transcriptional repressors^66^,^119^,^120^. The repressor for the SHH signaling pathway, Patched (Ptch1), is also found in the ciliary membrane, and inhibits pathway activation in the absence of ligand by repressing the downstream activator, SMO^67^. When SHH ligand is present, SHH binds Ptch1, causing it to move out of the cilium, and this allows SMO to enter the cilia^65^,^67^. SMO localization to the cilium inhibits GliR formation and, via an unknown mechanism, the full-length GLIs become GLI activators^121^. Precisely how SHH signaling proteins are targeted and moved in and out of the cilium is not clear, but intraflagellar transport (IFT) is required^66^,^120^,^122^. Anterograde IFT carries cargo toward the tip of the cilium, while retrograde transport carries turnover products out of the cilium; deletion of anterograde or retrograde IFT proteins results in distinct ciliary phenotypes. The neural tube defects and the stubby cilia morphology of EHD1-null embryos resemble the ciliary phenotype of mice that carry mutations in IFT122. In these mice, retrograde transport is impaired and GLI2 and SMO accumulate in the cilia even in the absence of SHH signaling. To better determine how SHH signaling is regulated by EHD1, we examined the dynamics of SHH components using antibodies against endogenous proteins. Normally, GLI2 localizes to the entire ciliary axoneme and is enriched at the ciliary tip after SHH stimulation^68^,^69^. Indeed, when we measured the fluorescence intensity in the tips of cilia relative to background staining using antibodies that recognize full-length GLI2, we found GLI2 that was enriched in cilia of wild-type MEFs after treatment with SAG-conditioned media (Fig. 10A) Interestingly, there was no significant change in GLI2 localization to the entire cilia under basal conditions and GLI2 enrichment at the ciliary tips after SHH pathway activation in EHD1-null MEFS (Fig. 10A). SMO and PTCH1 localize along the length of the cilium in a complementary manner: PTCH1 in the absence of ligand and SMO upon pathway stimulation^65^,^67^. As expected, we observed an increase in ciliary SMO levels after SHH stimulation in wild-type MEFs (Fig. 10B). Notably, in the absence of EHD1 we found SMO localized to the cilium in null MEFS without SHH stimulation (Fig. 10B) with further enrichment upon SHH stimulation (Fig. 10B) suggesting that EHD1 plays a role in regulating the entry of SMO into cilia in response to SHH pathway activation. Consistent with this idea, we found that EHD1 to co-localize with SMO in the WT MEF cilia after SHH pathway activation in 70% of cells counted (Fig. 11A). More direct support for a role of EHD1 in aiding the trafficking of SMO into primary cilia upon SHH pathway stimulation was provided by live-cell imaging of SMO-GFP stably expressing NIH3T3 in which a DsRed EHD1 was expressed (Fig. 11B). These studies demonstrated that under non-stimulated conditions EHD1 and SMO indeed co-localize at vesicular structures inside the cell-body, but once SHH pathway is activated with SAG, EHD1 and SMO co-trafficked into primary cilia (Fig. 11B). This observation led us to consider whether EHD1 directly associates with SHH pathway component SMO. Indeed, EHD1 showed co-immunoprecipitation with SMO-GFP (expressed at endogenous levels) in NIH3T3 cells (Fig. 12A). Furthermore, *in vitro* GST-pulldown assay utilizing GST-EHD1 and NIH3T3 cells stably expressing SMO GFP showed that EHD1 and SMO interact directly (Fig. 12B). Notably, failure of a GST-EHD1 fusion protein lacking the EH domain to pulldown SMO strongly supports a mechanism involving the binding of the EH domain of EHD1(Fig. 12B). The N-terminal G domain of EHD1 is required for ATP binding and membrane recruitment, the central region for dimerization/oligomerization, and the C-terminal EH domain for binding to NPF motifs in a number of proteins^18^ Analysis of the amino acid sequence of mouse and human SMO using UNIPROT database revealed a conserved NPF motif in the cytoplasmic tail of SMO.

Future studies using NPF/DPF domain mutant SMO will help further address if the mechanism proposed here is the basis for a the EHD1-SMO interaction. Further examination of the mechanism of EHD1 interaction with the ciliary proteome should also help assess if other players like Rab23, Sufu, Patched, Gpr161 and PKA may also interact, either directly or indirectly, with EHD1.

The apparent lack of spatial and temporal control of SHH expression and signaling in the absence of EHD1-regulated ciliary SHH signaling provides a plausible explanation for neural tube defects observed in EHD1-null embryos. The novel role of the endocytic recycling pathway regulator EHD1 in ciliary trafficking and signaling we describe here is consistent with emerging evidence implicating a number of vesicular trafficking proteins in primary ciliogenesis For example, the ENU-induced Rab23 mutations in mice revealed that Rab23 negatively regulates the SHH signaling pathway during neural tube formation^103^ and Rab23 mutant cells were shown to have an accumulation of SMO into the cilium^123^.

In conclusion, the results presents here suggest a previously undefined role of the endocytic regulator EHD1 in regulating activation and repression of the SHH pathway by regulating the trafficking of SHH pathway signaling protein SMO into the cilia in response to SHH activation. Emerging evidence in different model systems have demonstrated the role of vesicular trafficking in primary ciliogenesis, but this is the first report of EHD1, an endocytic recycling regulator to play a crucial role in ciliary trafficking of SMO and SHH signaling. Our results point to a role of EHD1 in the fine regulation of the balance between trafficking of SHH signaling proteins into and out of the cilium which in turn is critical for maintaining the correct ratio of GLI activators to GLI repressors and proper activation of the SHH pathway during development and other processes. Future studies examining the EHD1-associated proteome in this process should provide new insights into developmental as well as disease-associated roles of this protein family.

## Additional Information

**How to cite this article**: Bhattacharyya, S. *et al.* Endocytic recycling protein EHD1 regulates primary cilia morphogenesis and SHH signaling during neural tube development. *Sci. Rep.* **6**, 20727; doi: 10.1038/srep20727 (2016).

## Supplementary Material

Supplementary Information

## Figures and Tables

**Table 1:** EHD1 Deletion causes mid-gestation lethality.

*Gestation Days*	Ehd1+/+	Ehd1+/−	Ehd1−/−	Total
*E9.5*	26 (27)	59 (54)	23 (27)	108
*E10.5*	14 (15)	34 (29)	10* (14)	58
*E11.5*	10 (7)	20 (15)	0 (8)	30

The numbers of embryos corresponding to each genotype retrieved at various stages after *Ehd1*^+/−^ X *Ehd1*^+/−^ crosses are presented. The numbers within parentheses are those expected based on Mendelian ratios. The asterisk represents embryos showing signs of re-absorption at the time of isolation.
