# Corrigendum: Endocytic recycling protein EHD1 regulates primary cilia morphogenesis and SHH signaling during neural tube development

**DOI:** 10.1038/srep42320

**Published:** 2017-03-23

**Authors:** Sohinee Bhattacharyya, Mark A. Rainey, Priyanka Arya, Bhopal C. Mohapatra, Insha Mushtaq, Samikshan Dutta, Manju George, Matthew D. Storck, Rodney D. McComb, David Muirhead, Gordon L. Todd, Karen Gould, Kaustubh Datta, Janee Gelineau-van Waes, Vimla Band, Hamid Band

Scientific Reports 6: Article number: 2072710.1038/srep20727; published online: 02 17 2016; updated: 03 23 2017

A reader alerted us of a potential error in Figure 12B and its accompanying Supplementary Figure 13B, since the indicated bands for GST fusion proteins did not correspond to the distinct expected sizes. We have indeed found an error in the description of the GST constructs used in our paper and labeling of an apparently background band as GST fusion proteins. Here, we have provided a new set of figures to replace the ones included in the paper. We regret any confusion or inconvenience our error caused to the readers of our paper.

By DNA sequencing of the constructs used for the pulldown, we determined that the constructs designated as GST-EHD1 and GST-EHD1-ΔEH in fact corresponded to the EHD1 amino acids 399-534 (now designated GST-EHD1-399-534; includes the EH domain and a portion of the preceding helical region; constructed by cloning a PCR fragment in the pGEX2T vector) and 438–534 (now designated GST-EHD1-438-534; Addgene Plasmid #36459), respectively, fused at the N-terminus to GST. The latter includes the actual EH domain sequence, and the EHD1-436-534 fragment has been reported to form a functional EH domain *in vitro*^1^. We have carried out new pulldown analyses with these constructs as well as a GST-EHD1-399-534-W485A mutant expected to show markedly reduced binding to target proteins^2^,^3^.The experimental results (the corrected Figure 12B appears as Figure 1 and corrected Supplementary Figure 13B appears as Figure 2; these replace the original figures in our paper) confirm our previous conclusion that Smoothened protein interacts with the EH domain of EHD1. GST-EHD1-399-534 fusion protein strongly pulled down Smoothened while GST-EHD1-436-534 did not; notably, the W485A point mutation in the GST-EHD1-399-534 fusion protein markedly reduced the Smoothened pulldown, consistent with EH domain-mediated pulldown. Ponceau staining of the membrane used in the blot established the correct migration and equal loading of the fusion proteins. The lack of Smoothened pulldown with the GST-EHD1-438-534 construct suggested that this shorter construct may be less efficient at stable interaction under the conditions used. To assess if this is the case, we carried out pulldown experiments using a known target protein rabenosyn 5, whose multiple high-affinity NPF motifs mediate strong interaction with EHD1^1^,^4^. Pulldown of rabenosyn 5-GFP showed that the level of pulldown with GST-EHD1-438-534 was markedly lower compared to that with GST-EHD1-399-534, while the W485A mutant did not show binding. While the precise reasons for lower (rabenosyn 5) or absent (Smoothened) binding of target proteins to EHD1-438-534 construct remain unclear, a potential explanation may be the lack of the conserved preceding amino acids in this construct and/or the fusion to GST too close to the alpha13 helix^5^, which may distort the EH domain structure.

The results shown in the corrected figures confirm the conclusion of the originally-presented experiment, and help rectify our incorrect designation of the GST fusion proteins.

The list of authors has also been corrected to include two new authors (Bhopal C. Mohapatra and Insha Mushtaq) who performed the new experiments presented here. The corrected list and sequence of authors now reads: Bhattacharyya S., Rainey M.A., Arya P., Mohapatra B.C., Mushtaq I., Dutta S., George M., Storck M.D., McComb R.D., Muirhead D., Todd G.L., Gould K., Datta K., Waes J.G., Band V., Band H.

The author contribution statement now reads:

S.B. designed and performed experiments, analyzed data and wrote the first draft of the manuscript. M.R. made initial observations of embryonic lethality. P.A. provided technical help and suggestions for improvement. S.D. provided technical help, scientific advice and protocols. M.R., M.S. and M.G. helped generate and maintain the floxed mice. BCM and IM performed experiments included in the Correction. R.M., D.M. and G.T. provided technical assistance with the EM studies. K.G. provided assistance with the Dartmouse genetic background analysis. K.D. and J.W. provided scientific advice, protocols and suggestions for improvement. H.B. and V.B. conceived the study and secured funding. H.B. supervised the project, designed the experiments, analyzed data and edited the manuscript. All authors read the manuscript and provided feedback.

## Figures and Tables

**Figure 1:**
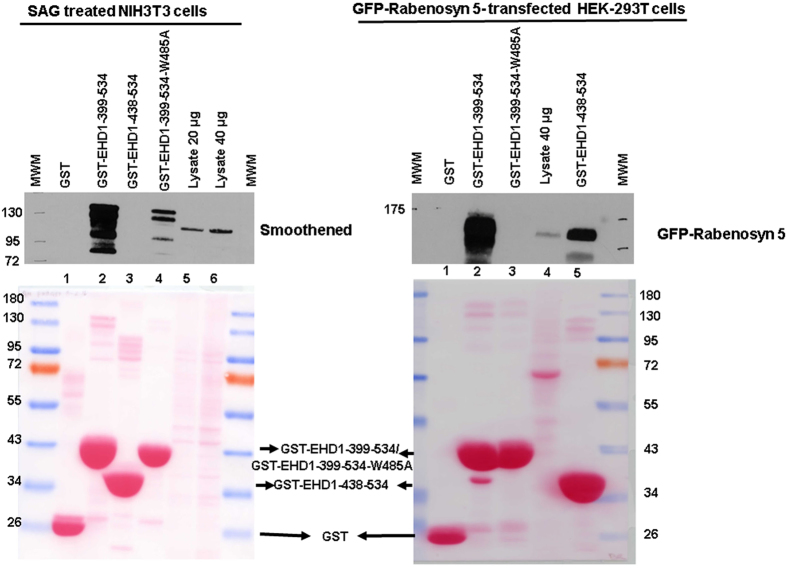
Left Panel: NIH-3T3 cells were serum-deprived for 24 hours and then stimulated with smoothened agonist (SAG) in the same media for another 24 hours prior to lysate preparation. 5 mg aliquots of cell lysate protein were used for pulldown with 50 μg of bacterially-expressed purified glutathione S-transferase (GST) (lane 1, control), GST-EHD1-399-534 (EHD1 amino acids 399 to 534 fused on the N-terminus to GST; lane 2), GST-EHD1-438-534 (EHD1 amino acids 438 to 534 fused to GST; lane 3) or GST-EHD1-399-534-W485A mutant (GST-EH1 construct with a W485A point mutation in the EHD1 sequence; lane 4) fusion proteins non-covalently bound to Glutathione-Sepharose beads. After washing, the bound proteins were visualized by anti-smoothened immunoblotting. The whole cell lysates (20 μg or 40 μg) were concurrently resolved (lane 5 and lane 6). The molecular weight markers (in KD) were run on both sides and are indicated on the left. Lower panel shows Ponceau S staining for 5 minutes prior to immunoblotting to visualize equal GST fusion protein amounts used in pulldown and their expected sizes: GST, ~26 KD; GST-EHD1-399-534 and GST-EHD1-399-534-W485A, ~41 KD; GST-EHD1-438-534, ~37 KD. The data is representative of three separate experimental repeats. **Right panel:** 2 mg aliquots of cell lysate protein from HEK-293T cells transiently transfected with a human Rabenosyn 5-GFP construct^6^ (Addgene Plasmid #37538) were used for pulldown as for the NIH-3T3 cell lysates above with 50 μg of bacterially-expressed purified glutathione S-transferase (GST as control; lane 1), GST-EHD1-399-534 (EHD1 amino acids 399 to 534 fused on the N-terminus to GST; lane 2), GST-EHD1-399-534-W485A mutant (GST-EH1 construct with a W485A point mutation in the key NPF motif contact residue; lane 3), GST-EHD1-438-534 (EHD1 amino acids 438 to 534 fused to GST; lane 5) fusion proteins non-covalently bound to Glutathione-Sepharose beads. After washing, the bound proteins were visualized by anti-GFP immunoblotting. The whole cell lysates (40 μg) were concurrently resolved (lane 4). Lower panel shows Ponceau S staining for 5 minutes.

**Figure 2:**
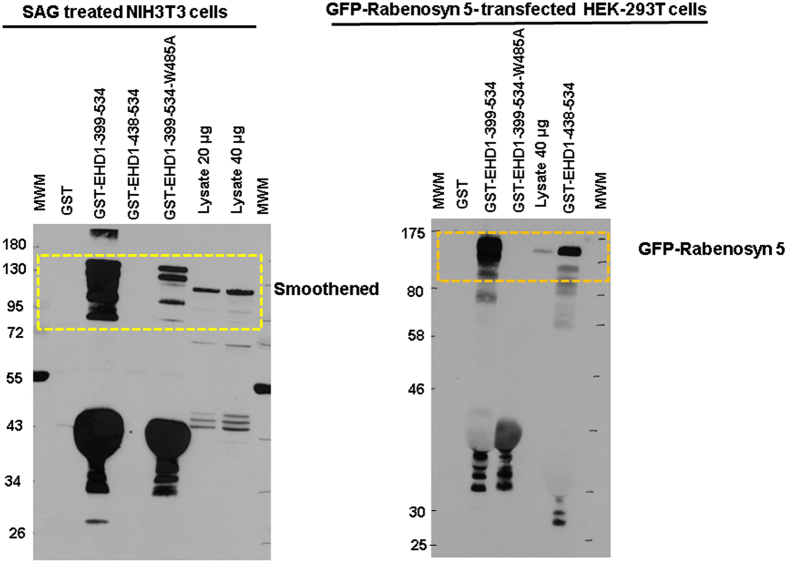
Left Panel: The entire anti-smoothened antibody blot used for the top portion of Fig. 12B (Corrected) is presented, with the cropped-out region indicated, and replaces the corresponding blot shown in the published paper. **Right Panel:** The entire anti-GFP blot from which the top portion of the right panel of Fig. 12B (Corrected) was generated is shown, with the cropped-out region indicated.
