# Supplementary material for: Endocytic recycling protein EHD1 regulates primary cilia morphogenesis and SHH signaling during neural tube development
Source: Sci Rep. 2016 Feb 17;6:20727. doi: 10.1038/srep20727 (PMC4756679; doi:10.1038/srep20727)
Supplement: Supplementary Information [file srep20727-s1.docx]

# SUPPLEMENTARY INFORMATION

**Endocytic recycling protein EHD1 regulates primary cilia morphogenesis and SHH signaling during neural tube development**

Sohinee Bhattacharyyaa,d, Mark A Raineyd, Priyanka Aryab,d , Samikshan Duttac, , Manju Georged, Matthew D Storckd, Rodney D McComba, David Muirheada, Gordon L Toddb, Karen Gouldb, Kaustubh Dattac, Janee Gelineau-van Waes f, Vimla Bandb, d, e and Hamid Banda, b, d, e

**Figure S1**


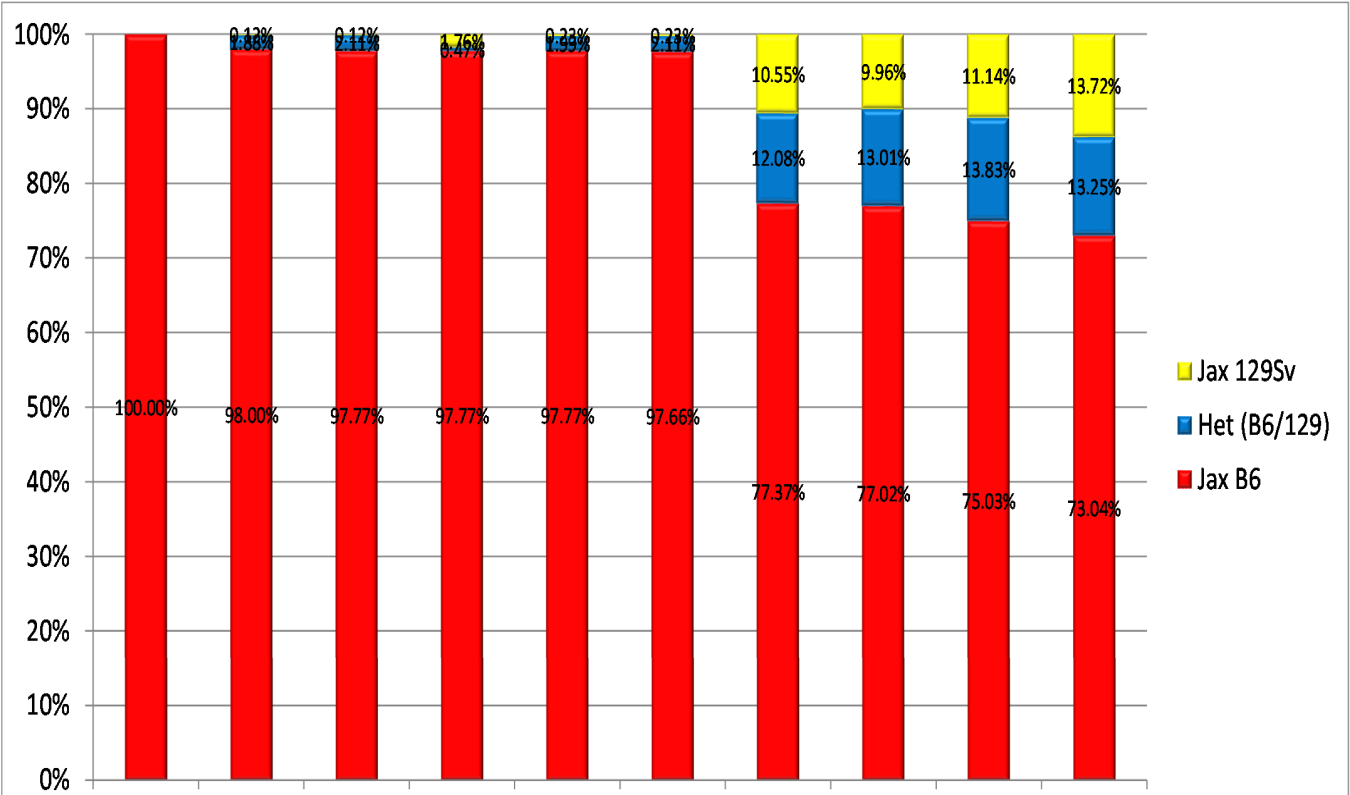


**Pure B6 control**

**N5B6 mice used in this study B6.129 mixed background mice**

**used in previous studies**

**Figure S1 Genetic Background Verification**

Tail snip DNA was analyzed for the extent of B6 genetic background by PCR-based typing of polymorphic loci throughout the genome (though The DartMouse Speed Congenic Facility at The Geisel School of Medicine at Dartmouth). A, pure B6 control from The Jackson Laboratory. B-F, representative *Ehd1+/-* male and female mice backcrossed four times to B6 mice, starting with mixed B6/129 background; ~98% B6 background. G-J, B6/129 mixed background mice used in our previous studies; 73-77% B6 background.

**Supplementary figure 1, Bhattacharyya *et al***

**Figure S2**

**A**


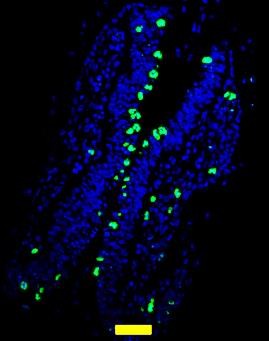
**WT EHD1 -/-**

**PH3-DAPI**

**PH3-DAPI**


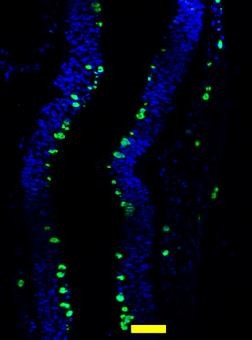


**B**

**WT EHD1 -/-**

**Cleav. Casp. 3 - DAPI**

**Cleav. Caspa.3 -DAPI**

**12**

**9**

**NS**

**POSITIVE NUCLEI**

**6**

**3**

**PH3**

**0**

**WT EHD1 -/-**


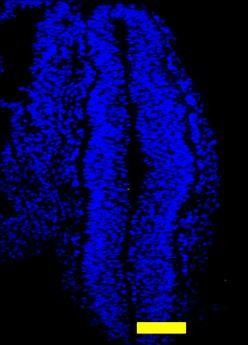

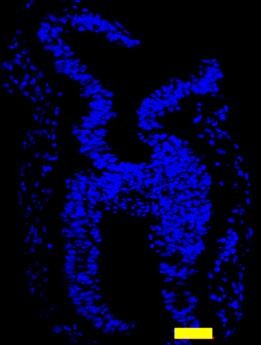


**Figure S2 Analysis of proliferation and apoptosis in the neuroepithelium of WT vs *Ehd1*- null embryos**

1. E9.5 WT control and *Ehd1-null* transverse neural tube sections immunostained for phospho- Histone-3 (pH3, green signal) and counterstained with 4,6-diamidino-2-phenylindole (DAPI, blue signal). Quantitation of proliferation in WT and *Ehd1*-null neuroepithelium shows no significant difference in the number of proliferating neuroepithelial cells in the neural tube. Scale Bar: 50 µm.
2. E9.5 WT and *Ehd1*-null transverse neural tube sections immunostained for the apoptosis marker Cleaved-Caspase3 (CC3) reveal no significance difference in levels of neuroepithelial cell- death between the WT and *Ehd1*-null embryo. Scale Bar: 50 µm.

**Supplementary figure 2, Bhattacharyya *et al***

**Figure S3**

**PECAM1-DAPI**

**WT EHD1 -/-**


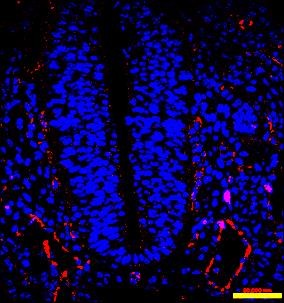


**DA**

**DA**


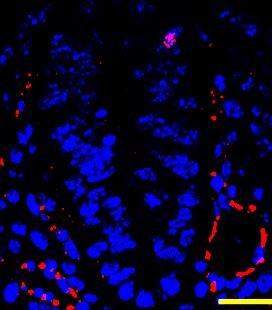


**DA**

**DA**


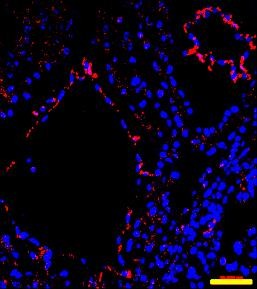


**HT**

**HT**


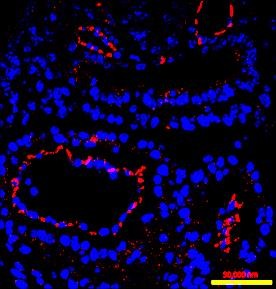


**HT**

**HT**


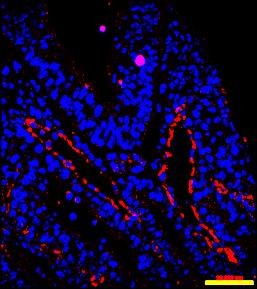


**DA**

**DA**


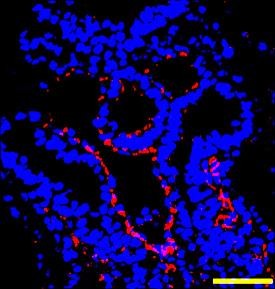


**DA**

**DA**

**Figure S3 Analysis of vasculogenesis in WT vs *Ehd1*-null embryos**

Transverse sections of E9.5 WT and *Ehd1*-null embryos stained with PECAM1 antibody showing endothelial cells in the dorsal aorta of the head, heart and dorsal aorta of the tail. No obvious defects were observed in vascular development in the *Ehd1*-null embryo as compared to the WT. Abbreviations: da, dorsal aorta; ht, heart. Scale Bar: 50 µm

**Supplementary figure 3, Bhattacharyya *et al***

**Figure S4**

**A B**

**WT EHD1 -/-**


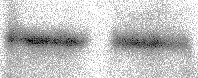

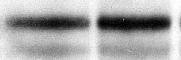

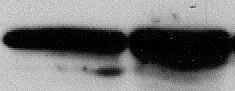

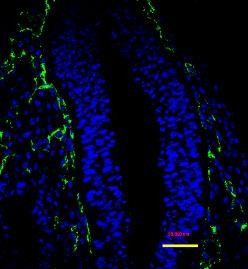

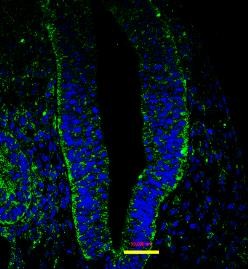

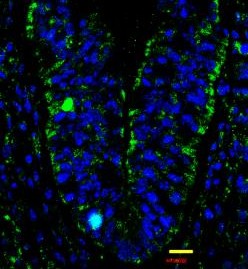

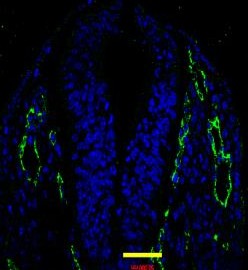
**WT EHD1 -/-**

**Dysferlin-DAPI**

**Myoferlin-DAPI**

**Dysferlin-DAPI**

**Myoferlin-DAPI**

**Dynamin I Clathrin H**

**AP2**

**HSC 70**

**Pacsin2 Epsin 1**

**SNAP 29**

**HSC 70**

|  |
| --- |
| 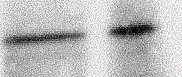 |
|  |
|  |

**WT EHD1 -/-**


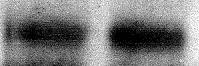

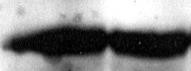

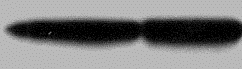

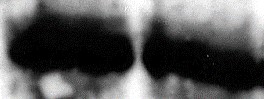


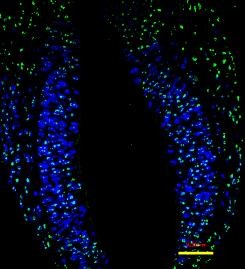

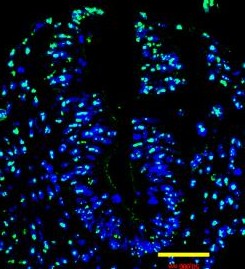
**Figure S4 Analysis of major endocytic partners of EHD1 in WT vs *Ehd1*-null embryos**

**SNAP29-DAPI**

**SNAP29-DAPI**

1. Expression of endocytic proteins that interact with EHD1 as revealed by biochemical analysis of pooled whole-embryo lysates at E9.5. No obvious difference in expression levels was observed between WT and *Ehd1*-null embryos.40 µg of protein from pooled embryo lysates from E9.5 WT control and *Ehd1*-null embryos were separated using 8% SDS-PAGE and immunoblotted using antibodies against Dynamin I, Clathrin H, AP2, Pacsin2, Epsin1 and SNAP29. HSC-70 is the loading control.
2. E9.5 WT and *Ehd1*-null transverse neural tube sections immunostained for major endocytic proteins that interact with EHD1. No obvious difference in expression pattern was observed between the WT and *Ehd1-null* neural tubes.

**Supplementary figure 4, Bhattacharyya *et al***

**Figure S4C. Analysis of major endocytic partners of EHD1 in WT vs EHD1-null MEFS**

**WT MEF EHD1 -/- MEF**

**Pacsin2**


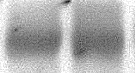


**Clathrin**


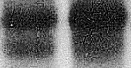


**Clathrin**

**Dynamin Dynamin**


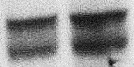


**AP2**


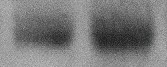


**HSC70**


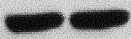


Expression of endocytic proteins that interact with EHD1 as revealed by western blot analysis of control WT MEFS and EHD1-null MEFS. No obvious difference in expression pattern was observed between WT and *Ehd1*-null MEFS.

**Figure S4D. Analysis of major endocytic partners of EHD1 in WT vs**


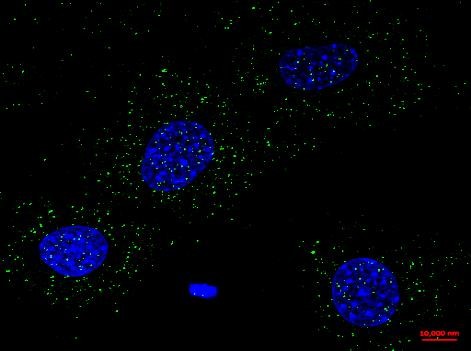


**Dynamin-DAPI**

**Pacsin1-DAPI**

**Pacsin1-DAPI**

**Dynamin-DAPI**

**EHD1-null MEFS**

**WT EHD1 -/-**


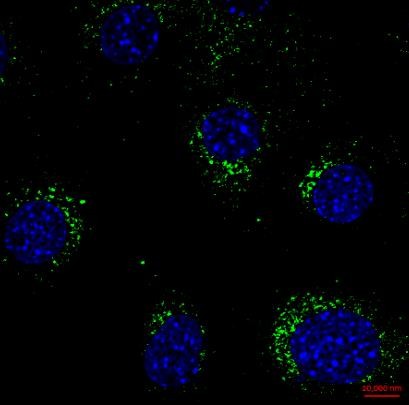

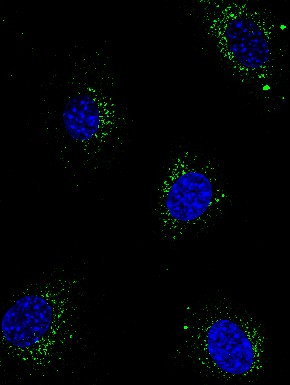


**Clathrin H-DAPI**

**Clathrin H-DAPI**


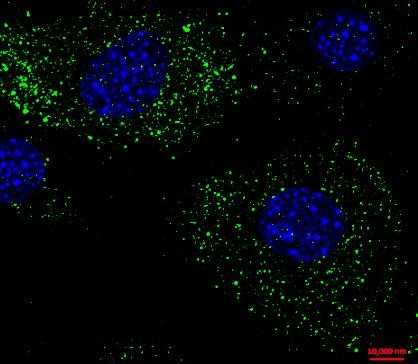

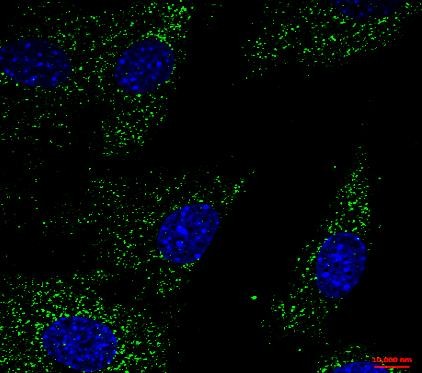

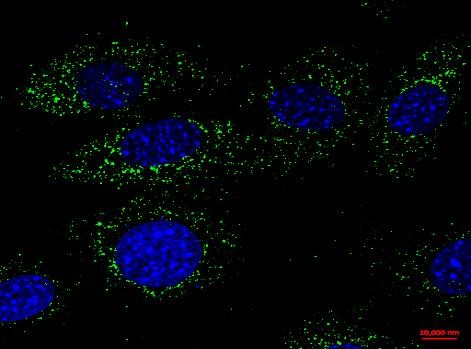

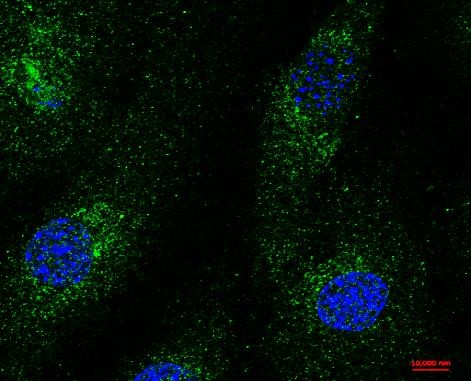


Expression of endocytic proteins that interact with EHD1 as revealed by immunofluorescence analysis of control WT MEFS and EHD1-null MEFS. No obvious difference in expression pattern was observed between WT and EHD1-null MEFS. Scale Bar:10 µm for WT MEFS and 20 µm for EHD1-null MEFS


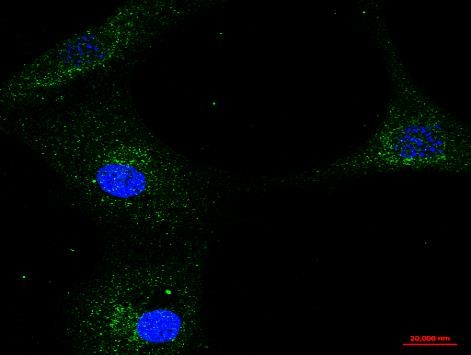


**Epsin2-DAPI**

**Epsin2-DAPI**

**Figure S5. Analysis of proliferation of EHD1 WT and EHD1-null MEFS**


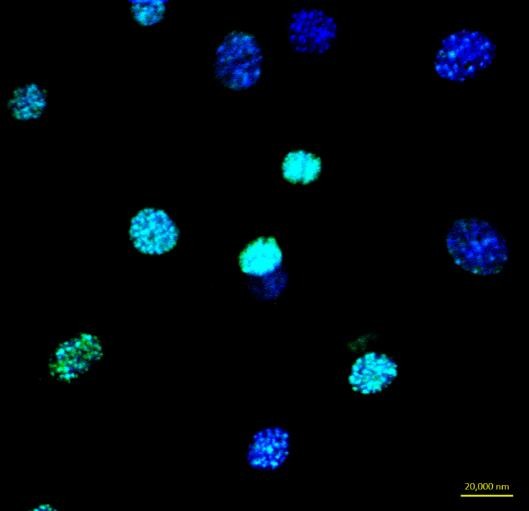

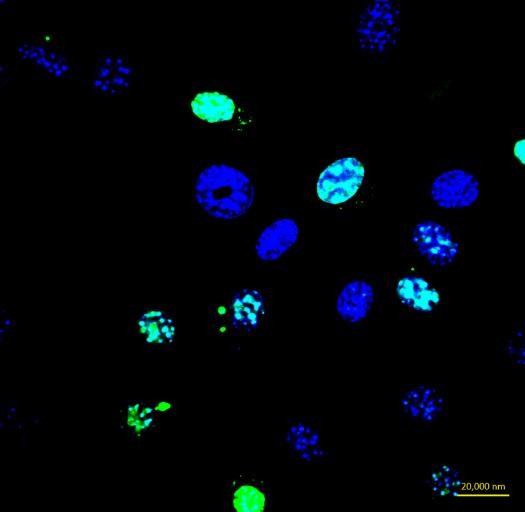
**WT EHD1 -/-**

**100**

**% Ki67 positive cells**

**75**

**50**

**25**

**0**

**WT EHD1 -/-**

******

p<0.05

E9.5 WT control and *Ehd1*-null MEFSwere immunostained for phospho-Histone-3 (pH3, green signal) and counterstained with 4,6-diamidino-2-phenylindole (DAPI, blue signal). Quantitation of proliferation in WT and *Ehd1*-null MEFS shows no significant difference in the number of proliferating cells upon EHD1 depletion.

**Figure S6**

**A**

**Shh-DAPI Foxa2-DAPI Nkx2.2-DAPI**


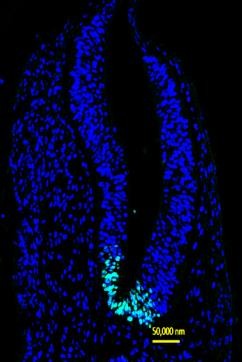

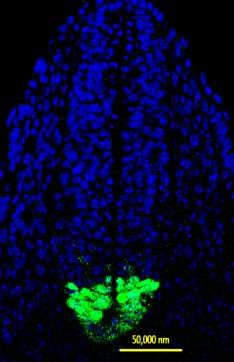

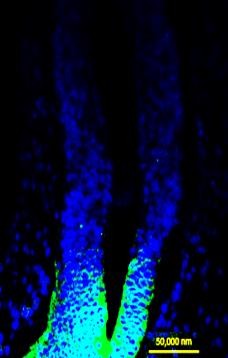


**Nkx6.1-DAPI**

**Pax6-DAPI Pax7-DAPI**


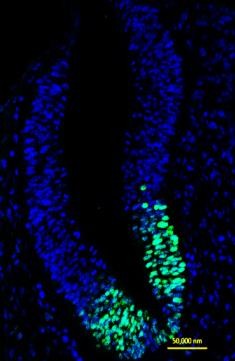


**WT**

**WT**

**EHD1 -/-**

**EHD1 -/-**


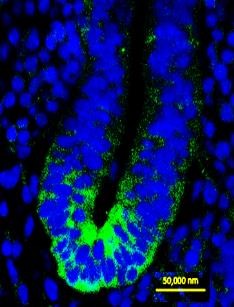

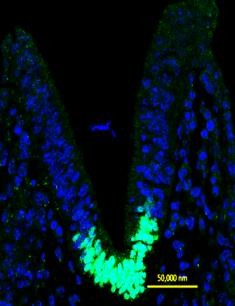

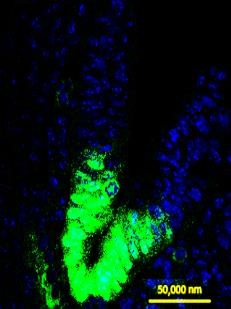

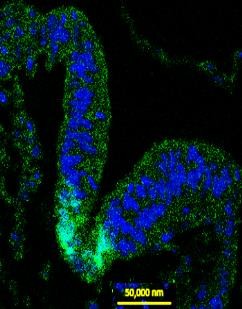

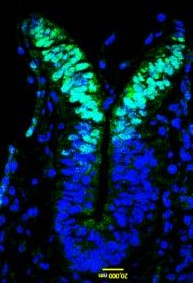

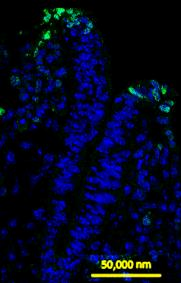

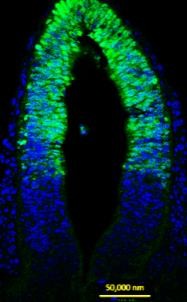

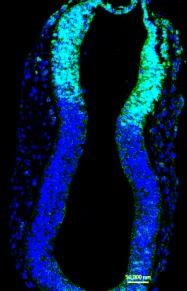


**B**

**EHD1 -/-**

**EHD1 -/-**

**Shh-DAPI Foxa2-DAPI Nkx2.2-DAPI Nkx6.1-DAPI**

**Pax6-DAPI Pax7-DAPI**


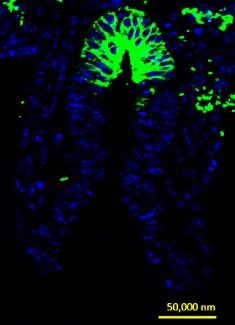

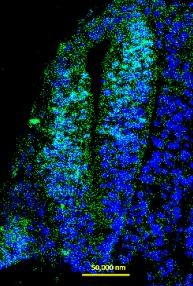

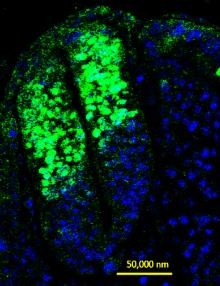

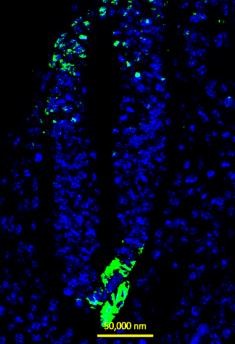

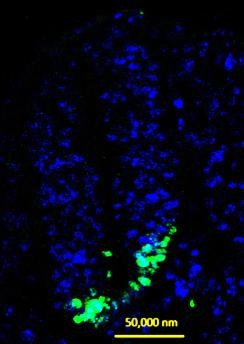

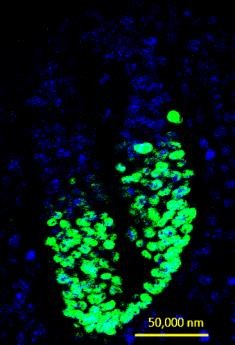

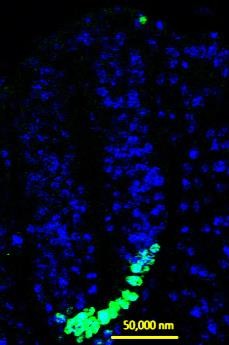


**WT**

**WT**

**Supplementary figure 6, Bhattacharyya *et al***


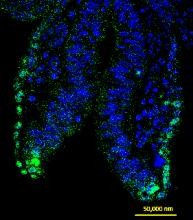

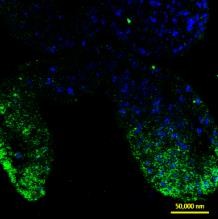

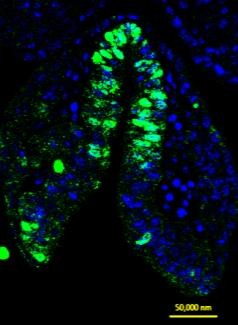

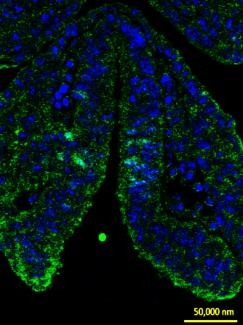

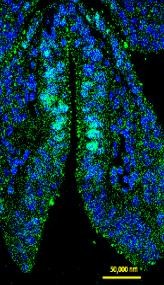


**Figure S6 Analysis of SHH specified neuronal patterning in WT vs *Ehd1*-null embryos**

1. E9.5 WT control and *Ehd1-null* embryo transverse sections of the neural tube (at the cranial level) were co-stained with antibodies against dorsal neuronal domain markers Pax6, Pax7 and ventral neuronal domain markers Nkx2.2, Foxa2 and Nkx6.1. In the *Ehd1-null* neural tube, expression of dorsal cell identitymarkers (Pax6 & Pax7) is severaly restricted, whereas the expression of ventral identy markers (Foxa2, Nkx2.2 and Nkx6.1) is expanded. Expression of SHH was unchanged in the *Ehd1-null* compared to the WT control littermate neural tubes. DAPI stains the nucleus. Scale Bar: 50 µm.
2. E9.5 WT control and *Ehd1-null* embryo transverse sections of the neural tube (at the lumbar level) were co-stained with antibodies against markers of dorsal and ventral neuronal domains as in A with identical observations. Scale Bar: 50 µm

**FigureS7. EHD protein expression during mouse embryonic development**


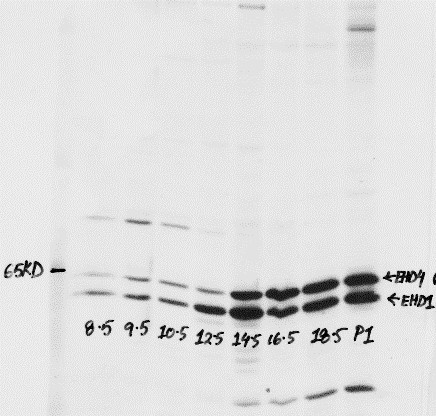


**EHD1&4**


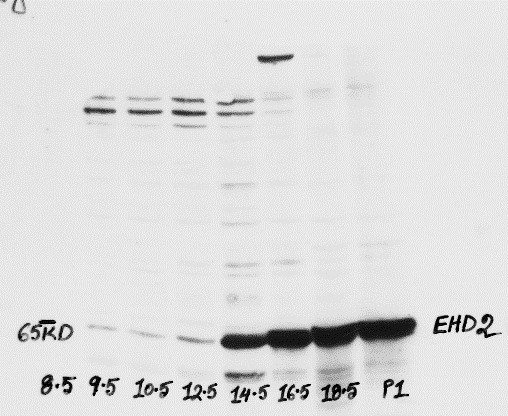


**EHD2**

**HSC70**


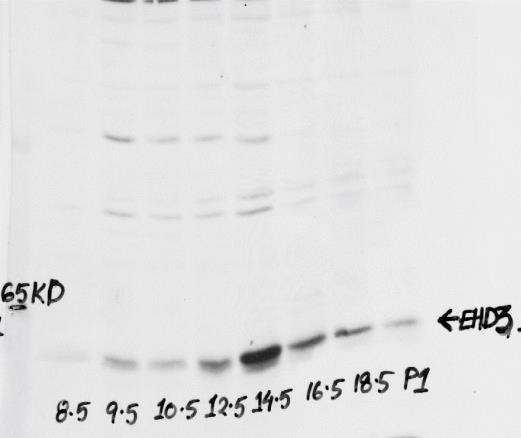


**EHD3**


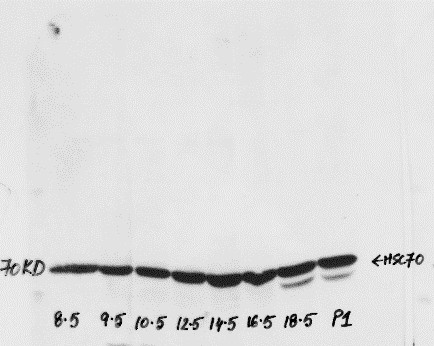

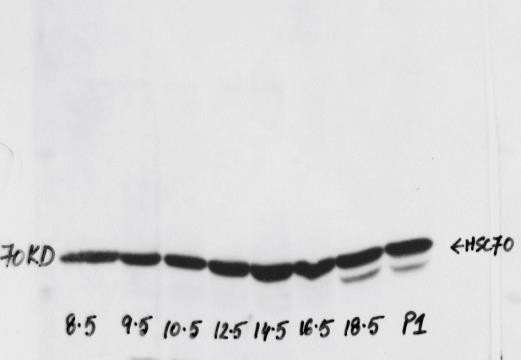


**HSC70**


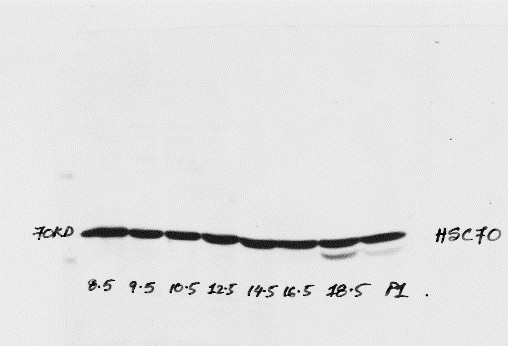


**HSC70**

Expression of EHD1, 2, 3 and 4 during embryonic development as revealed by western blotting of whole embryo lysates at the designated embryonic time points (E) and from total post-natal (P) fetal lysates of wild type mice. The EHD1/EHD4 membrane was serially stripped and re-probed with EHD2 antibody and a separate membrane was probed for EHD3. HSC 70 is the loading control. A single antibody recognizes both EHD1 and EHD4.

**FigureS8.Expression and localization of EHD proteins in WT and**

***Ehd1*-null embryos**


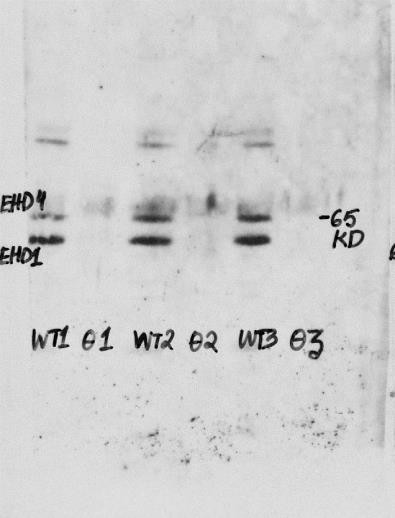


**EHD1&4**


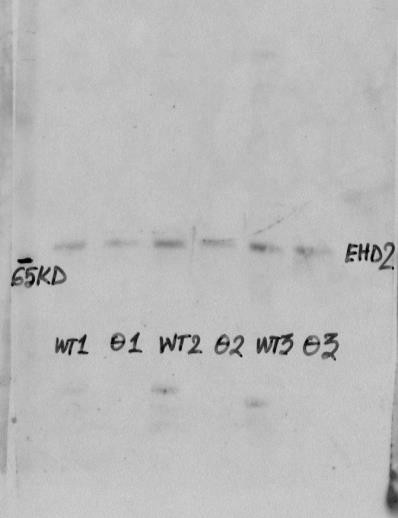


**EHD2**


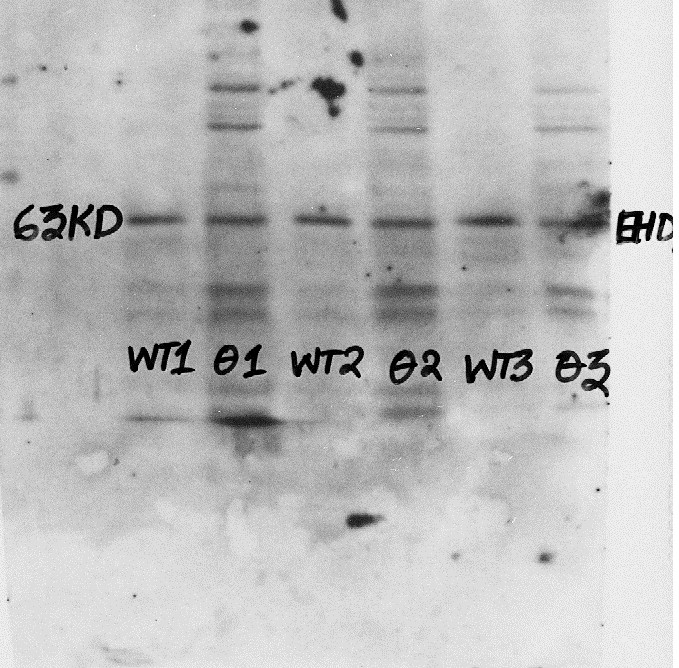


**EHD3**


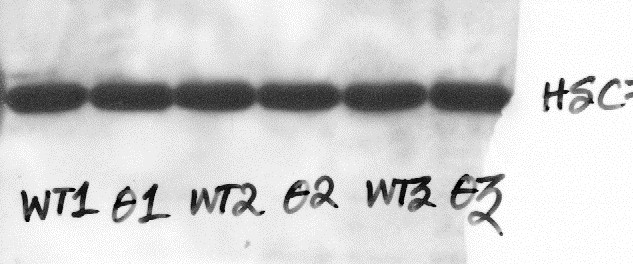

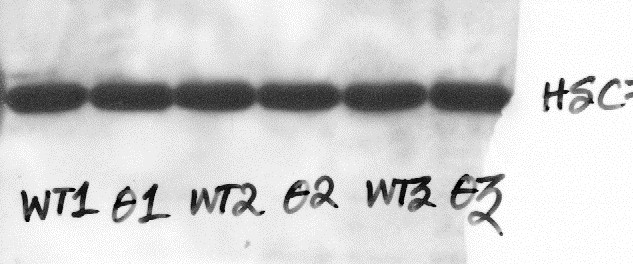


**HSC70**

**HSC70**


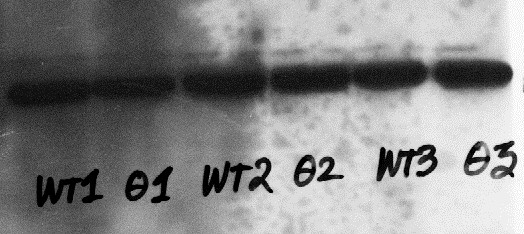


**HSC70**

40 µg aliquots of pooled E9.5 WT and *Ehd1*-null whole embryo lysate protein were separated using 8% SDS-PAGE and immunoblotted using rabbit antibodies against EHD1, EHD2, EHD3 and EHD4. HSC-70 is the loading control. The blot represents three individual experiments.

**FigureS9. *Ehd1*-null embryos have increased SHH signaling**

**GLI1 GLI2**


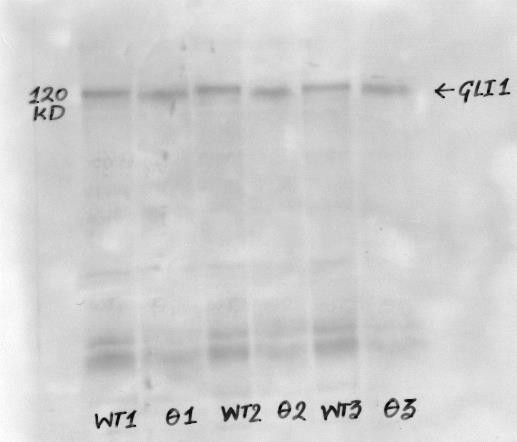

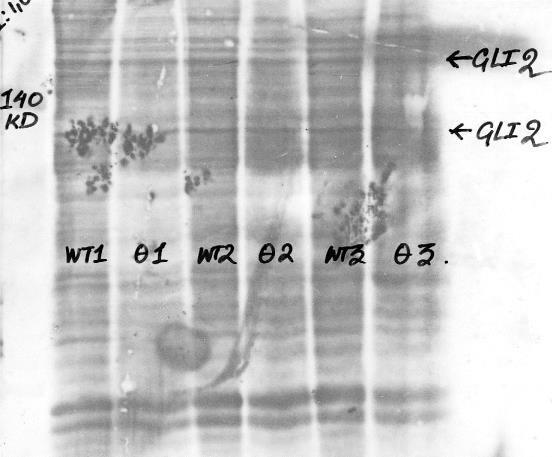

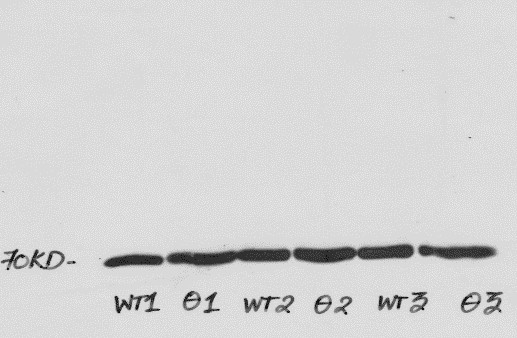


**HSC70**

**GLI3**


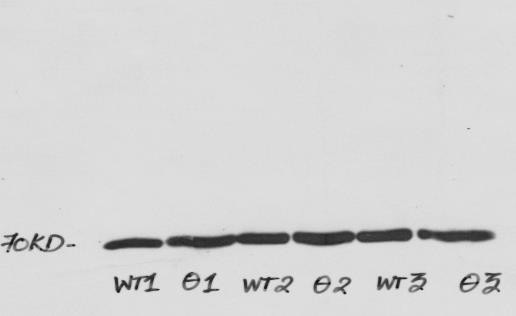


**HSC70**


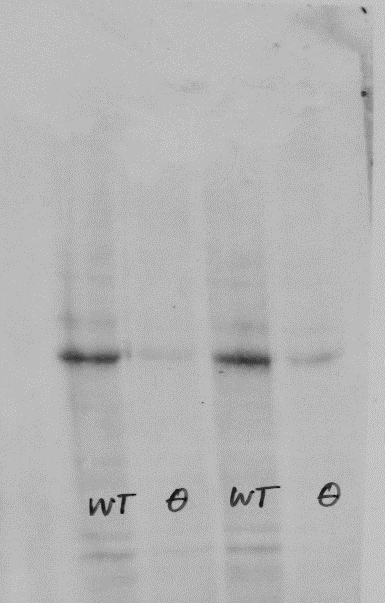


**85 KD**

**HSC70**


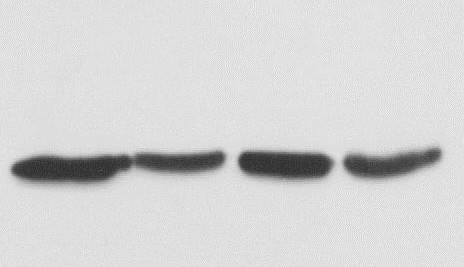


**70 KD**

# FigureS9. *Ehd1*-null embryos have increased SHH signaling

40 µg aliquots of pooled E9.5 WT and *Ehd1*-null whole embryo lysate protein were separated using 8% SDS-PAGE and immunoblotted using rabbit antibodies against GLI1,GLI2 and GLI3. HSC-70 is the loading control. The blot is representative of three individual experiments .

**Figure S10. Analysis of Ptch1 mRNA levels in the *Ehd1*-null and WT embryos**


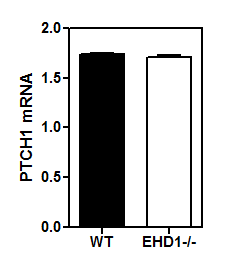


NS

Relative mRNA levels of Patched1 in the *Ehd1*-null and WT embryos measured by qRT-PCR analysis. Ptch1 mRNA levels remain comparable between *Ehd1*-null and WT embryos.

Unpaired t test; n=3 for each condition.

**Figure S11. Analysis of EHD protein expression in WT Vs EHD1-null MEFS**

**EHD4 EHD1**


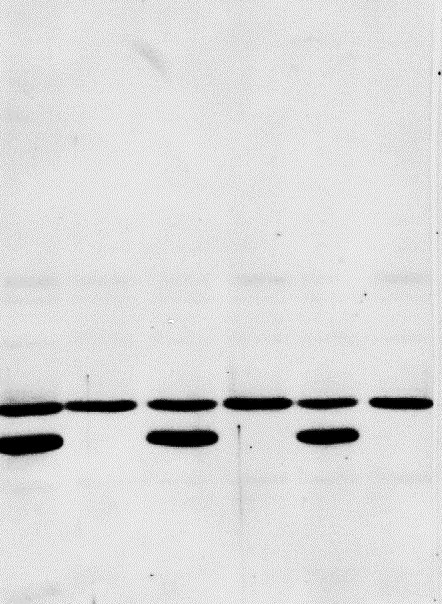

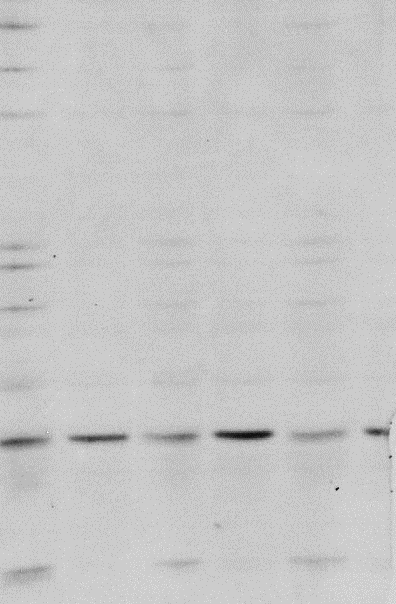


**EHD3**


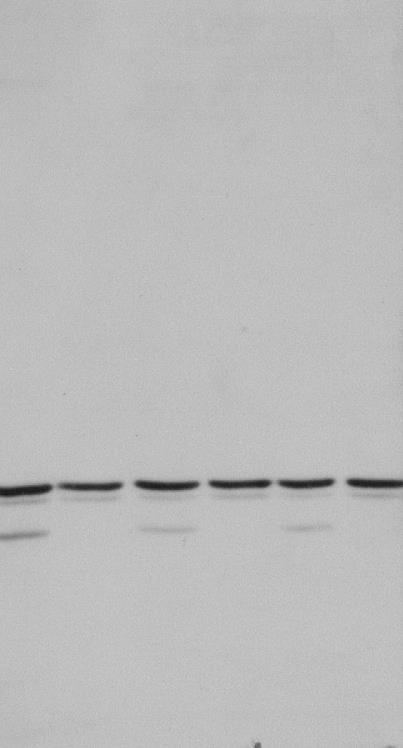


**EHD2**


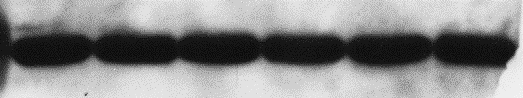

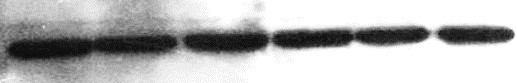

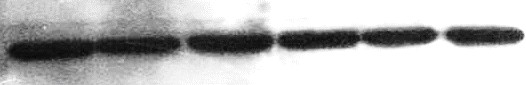
**HSC70**

**WT**

**NULL**

**WT**

**NULL**

**WT**

**NULL**

**WT**

**NULL**

**WT**

**NULL**

**WT**

**NULL**

**WT**

**NULL**

**WT**

**NULL**

**WT**

**NULL**

40 µg aliquots of WT and *Ehd1*-null MEF whole cell lysate protein were separated using 8% SDS-PAGE and immunoblotted using rabbit antibodies against EHD1, EHD2, EHD3 and EHD4. HSC-70 is the loading control.

**FigureS12. EHD1-null MEFS have decreased GLI3R expression levels upon SHH activation**

**-SAG +SAG**


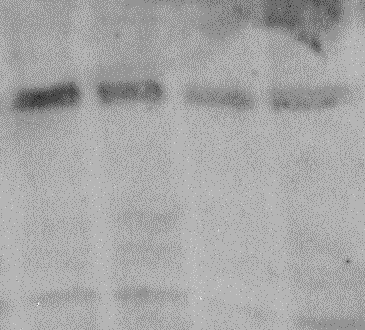

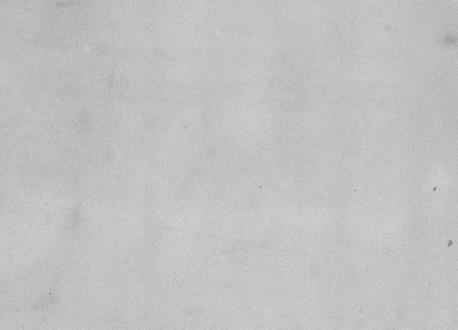


**GLI1**

**GLI1 120 KDa**


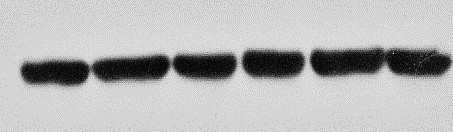

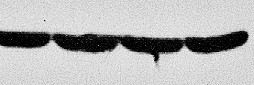
**HSC70 HSC70**

WT NULL WT NULL


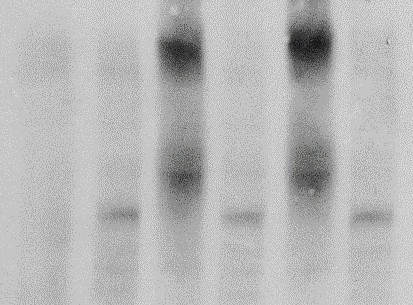

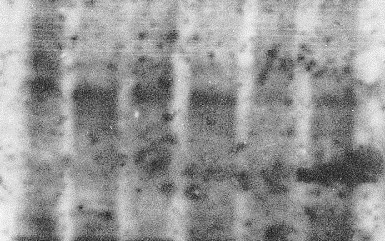


WT NULL

WT NULL

WT NULL

**GLI2**

**GLI2**

**140 KDa**

**HSC70**


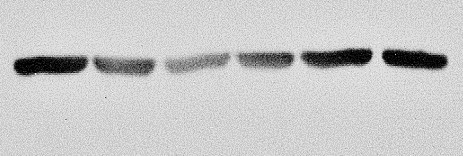


WT NULL WT NULL WT NULL


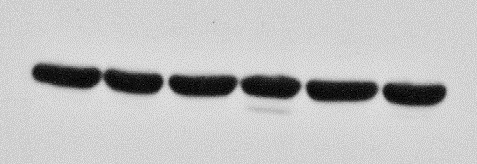


WT NULL WT NULL WT NULL

**HSC70**

**-SAG +SAG +SAG (repeat 2)**


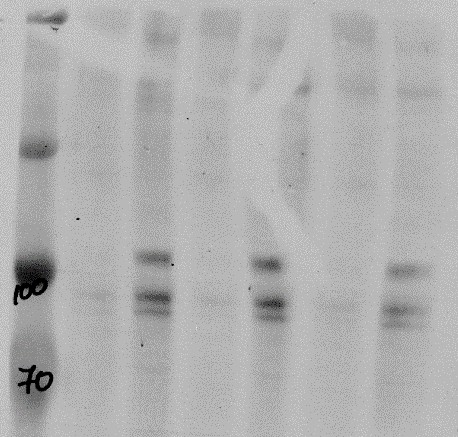

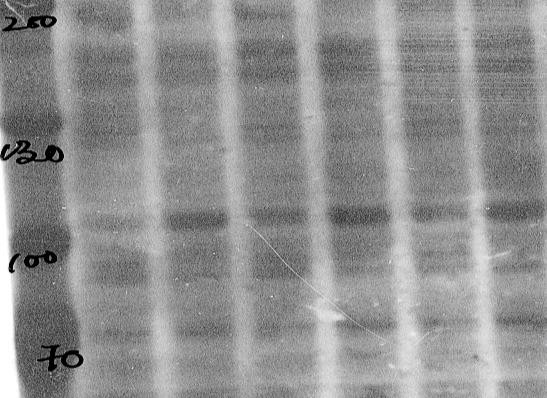


**GLI3 FL**

**GLI3 FL**

**GLI3 R**

**GLI3 R**

**175 KDa**


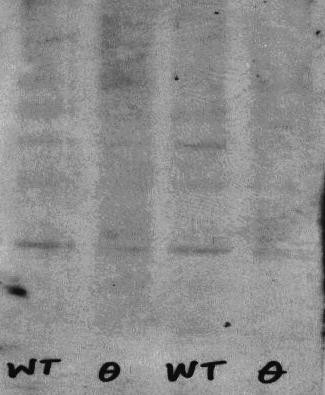


**GLI3 R**

**85 KDa**

**85 KDa**

**HSC70**

WT NULL

WT NULL

WT NULL

WT NULL WT NULL WT NULL

WT NULL

WT NULL

**HSC70**

40 µg aliquots of whole cell lysate protein from pooled WT and *Ehd1*-null MEFS under basal and SAG stimulated conditions were separated using 8% SDS-PAGE and immunoblotted using antibodies against GLI1, GLI2 and GLI3. HSC-70 is the loading control.

**FigureS13A. Immunoprecipitation assay reveals SMO to be a novel binding partner of EHD1**

**L**

**INPUT**

**IgG CT**

**IP:SAG**

**86 KDa SMO**

**65 KDa**

**EHD1**

Immunoblotting was used to assess the presence of SMO in anti-EHD1 immunoprecipitates from WT NIH3T3 cells stably expressing SMO GFP treated with SAG (24 hrs). Input samples show the amount of each protein in the whole extract before the IP. Immunoblots showing the amount of

SMO that co-precipitated with EHD1 from cells that stably expressed the

**FigureS13 B. EHD1 binding to SMO is mediated through the EH**

**domain of EHD1**

**INPUT**

**GST-**

**empty vector**

**IP:GST- EHD1**

**IP:GST-**

**del EH domain**

**86 KDa**

**65 KDa**

**SMO**

**GST**

WT NIH3T3 cells stably expressing SMO-GFP were starved in low serum media for 24 hours and stimulated with SAG in starvation media for another 24 hours and lysed and these lysates were incubated with GST-EHD1 or GST-EH domain deleted EHD1.The membranes were probed with antibodies to Smoothened and GST.GST-fused to the empty plasmid vector was used as a negative control.

**FigureS14. Staging of E9.5 WT Vs EHD1-null embryos**

**WT NULL**

**NT closure**

The mouse neural tube first fuses at the hindbrain/cervical boundary, at embryonic day (E) 8.5, creating two regions of active neural tube closure: rostrally within the hindbrain, and caudally in the upper spinal region. Subsequent closure in the mouse brain involves two further sites of fusion initiation and two additional neuropores (Golden and Chernoff, 1993; Fleming and Copp, 2000). We analyzed the number of somites in the *Ehd1*-null embryos and found the null embryos to have 14-18 somites in comparison to an average of 25 somites in the WT embryos. In the *Ehd1*-null embryos analyzed, the neural tube was closed from a point opposite the outflow tract to the proximal part of the tail (shown inside the bracket).
